# Liquid-Phase Synthesis of Monodispersed V^5+^ Faradic Electrode Toward High-Performance Supercapacitor Application

**DOI:** 10.3390/nano15161252

**Published:** 2025-08-14

**Authors:** Sutharthani Kannan, Chia-Hung Huang, Pradeepa Stephen Sengolammal, Suba Devi Rengapillai, Sivakumar Marimuthu, Wei-Ren Liu

**Affiliations:** 1 #120, Energy Materials Lab, Department of Physics, Science Block, Alagappa University, Karaikudi 630003, Tamil Nadu, India; sutharthanikannan26@gmail.com (S.K.); pradeepastephen8497@gmail.com (P.S.S.); 2 Department of Electrical Engineering, National University of Tainan, No. 33, Sec. 2, Shulin St., West Central District, Tainan City 700, Taiwan; chiahung@mail.mirdc.org.tw; 3 Metal Industries Research and Development Centre, Kaohsiung 81160, Taiwan; 4 Department of Chemical Engineering, R&D Center for Membrane Technology, Chung Yuan Christian University, 200 Chung Pei Road, Taoyuan 32023, Taiwan

**Keywords:** liquid-phase synthesis, V_2_O_5_, glycol, supercapacitors

## Abstract

Layered intercalating V_2_O_5_ (vanadium pentoxide) is a durable battery-type electrode material exploited in supercapacitors. The advancement of V_2_O_5_ nanomaterials synthesized from non-aqueous organic solvents holds significant potential for energy storage applications. Liquid-phase synthesis of orthorhombic V_2_O_5_ cathode material corroborated its compatibility with quartet glycols and allowed examination of their explicit roles in faradic charge storage efficacy. V_2_O_5_ was found to be an intercalative material in all the quartet glycols. The crystalline, rod-like morphology and monodisperse V_2_O_5_ electrode were ascribed to the effects of ethylene, diethylene, triethylene, and tetraethylene glycols. Notable differences were observed in the electrochemical analysis of the prepared V_2_O_5_ (EV, DV, TV, and TTV). In a three-electrode cell setup, the DV electrode demonstrated a superior specific capacity of 460.2 C/g at a current density of 1 A/g. From the Trasatti analysis, the DV electrode exhibited 961.53 C/g of total capacitance, comprising a diffusion-controlled contribution of 898.19 C/g and a surface-controlled contribution of 63.34 C/g. The aqueous asymmetric device DV//AC exhibited a maximum energy density of 65.72 Wh/kg at a power density of 1199.97 W/kg. The glycol-derived electrodes were anticipated to bepromising materials for supercapacitors and have the potential to meet electrochemical energy needs.

## 1. Introduction

Energy is the fundamental source of all major technological advancements. With the global shift toward clean energy, there has been a substantial rise in the construction of renewable energy power plants. Batteries and supercapacitors are of paramount importance in enabling a sustainable energy future by providing reliable and efficient energy storage capabilities. The maturing of supercapacitor technology has had a prodigious impact on the evolving electronic market and reduced the significance of energy paucity [1,2,3]. The rapidly increasing use of active material in nanoplatforms gained momentum in 2012, falling within the dimension range of (≤100 nm), and opened the door for broad applications in contemporary fields. Based on the appreciable characteristics, the nanoscale materials enhance the ionic conductivity and adequate storage capacity to accommodate all sites within a volume of material. Also, they exploit the various charge storage mechanisms, like surface-based ion adsorption, pseudo capacitance, and diffusion-limited intercalation processes. For this reason, nanoscale materials have assumed top priority in relation to the concerns of electrochemical energy storage [4,5,6]. Access to high-performance electrode material is decisive for mitigating energy shortages, as an electrochemical device would be nothing without the vigorous involvement of electrode materials. Particles of fine and non-agglomerated, homo-dispersed morphology are needed for the enhanced electrochemical application of various energy storage devices. However, achieving homogeneous dispersion of active materials remains a significant challenge in many synthesis processes. The quest for monodisperse morphology has given great propulsion to breakthroughs in polyol synthesis (liquid-phase synthesis), and the interest in polyol synthesis has increased for the synthesis of the nanostrip, nanocube, nanowire and microsphere morphology of active materials. Odorless, less toxic, and nonvolatile, glycols (diols) such as ethylene glycol (EG), diethylene glycol (DEG), triethylene glycol (TEG) and tetraethylene glycol (TTEG) are the cornerstones of liquid-phase synthesis. Their high dielectric constant tends to function as a solvent for the starting material and circumvent traces of byproducts. Glycols effectively reduce the metal particles of metal colloids in the precursor through heating near the boiling point and playing a multipronged role in the formation of metal nanoparticles. Precise control of the temperature and time was significant for the homogeneous formation of metal atoms from the precursor material [7,8,9,10,11]. The foremost report on nanoparticles from polyol synthesis was published by Fievet in 1989. He speculated on the detailed synthesis of nickel and cobalt compounds in a mixture of ethylene and diethylene glycols and offered a precedent for polyol synthesis. Subsequent reports on polyol synthesis have placedgreat emphasis on the development of nanoparticles via a non-aqueous route [12,13].

Critical research into supercapacitors involves evaluating criteria such as the enhanced electrochemical performance, extended cycle life, and superior energy and power capabilities of electrode materials by integrating nanostructuring strategies with carbon content enhancement. To date, redox-active electrode materials have undergone rigorous evolution with respect to specific capacities and cyclic stabilities. In a bid to identify a high-performance electrode material, V_2_O_5_ was initially known for its layered structure, multiple valences, and huge potential in supercapacitors. Yellow V_2_O_5_ is the stable form of the vanadium compound, and the availability of vanadium in the Earth’s crust (150 ppm), compared to other metal oxides, makes it affordable and aids the intense research on the application of the material in various electrochemical devices [14,15,16]. There is no astonishment that these vanadium materials have been perpetually investigated by researchers for supercapacitor application. Shireesha et al.’s group explored the effect of the hybridization of V_2_O_5_ with NiO and graphene using a one-pot combustion method. The electrode exhibited a superior specific capacitance of 936 F/g. The addition of graphene resulted in low enrichment of the prepared nanocomposite, which exhibited decreased charge transfer resistance. The NiO/V_2_O_5_@Graphene nanocomposite retained 89.90% of its capacitance after 3000 cycles at 100 mV/s. These results reveal that V_2_O_5_ benefits from hybridization in terms of the specific capacitance improvement [17]. Rohith and coworkers analyzed the electrochemical behaviors of V_2_O_5_ nanostructures in different neutral electrolytes, synthesized by a microwave-assisted method. These nanostructures exhibited superior performance in a KCl electrolyte and a specific capacitance of 82.35 F/g in a symmetric configuration with Na_2_SO_4_ electrolyte, demonstrating a retention efficiency of 70% after 10,000 cycles at 5 A/g. When fabricated as an asymmetric supercapacitor in a KCl electrolyte, it demonstrateda specific capacitance of 34 F/g [18]. Leistenschneider et al. fabricated carbon fiber (CF)-coated V_2_O_5_ with a high surface area of 440 m^2^/g using a dipping method. Asphaltenes were exploited as the carbon source. The electrode achieved a specific capacitance of 125 F/g at 0.25 A/g and exhibited 89% capacity retention after 10,000 consecutive cycles in a 1 M Li_2_SO_4_ electrolyte [19]. Bulla’s research group proposed three distinct V_2_O_5_ nanostructures as pseudocapacitive electrode materials using the same precursor source, NH_4_VO_3_. The resultant V_2_O_5_ synthesized at 180 °C, with a surface area of 38.6 m^2^/g, achieved a maximum specific capacitance of 178.5 F/g at 1 A/g in an aqueous electrolyte. In an asymmetric fabrication of V_2_O_5_//f-CNT, the device revealed an energy density of 37.12 Wh/kg and a power density of 800 W/kg at 1 A/g. The device retained 91.2% of its capacitance after 2000 charge–discharge cycles, attributed to the synergistic amalgamation of V_2_O_5_ (faradic reactions) and f-CNTs (electrical double-layer capacitance) [20]. The consistent application of V_2_O_5_ as a pseudocapacitive electrode material in supercapacitors has been widely documented, along with its superior characteristics. Thakur and his group carried out the insitu polymerization of anilinism hydrochloride to prepare a V_2_O_5_/PANI nanocomposite using the sol-gel method. The nanoflake assembly, with an optimized ratio of 20 wt.% V_2_O_5_/PANI, exhibited a substantial specific capacitance of 820.5 F/g. After 1000 cycles, it retained 88% of its initial capacitance at a current density of 1 A/g. In a symmetric assembly, it demonstrated an energy density of 4.6 Wh/kg and a power density of 80.7 W/kg. Additionally, the symmetric device highlighted its potential for energy storage applications by successfully illuminating a red LED in a series connection [21]. Temam et al. analyzed the supercapacitive performance of V_2_O_5_ electrode materials synthesized using different methods, including hydrothermal, solution combustion, and sol-gel techniques. These synthesis routes resulted in distinct morphologies: irregularly arranged nanosheets (hydrothermal), spherically agglomerated particles (solution combustion), and nanocubic shapes (sol-gel). Among the three, V_2_O_5_ synthesized via solution combustion exhibited the highest specific capacitance of 49 F/g and remarkably retained 108.4% of its initial capacitance after 5000 cycles at a current density of 1 A/g [22]. Singh et al. successfully synthesized ultralongV_2_O_5_ nanoribbons using commercial V_2_O_5_ and Pluronic P123 as precursor materials. The resultant nanoribbons possessed an average length of approximately 5–6 µm. The constructed asymmetric device, with activated carbon (AC) as the anode, demonstrated a specific capacitance of 65 F/g at a scan rate of 5 mV/s and retention of 77% after 1000 cycles. The V_2_O_5_-nanoribbon-based device exhibited an energy density of 17 Wh/kg at a power density of 1200 W/kg [23]. The synthesis of V_2_O_5_ nanorods for supercapacitors is a topic of significant interest. Previously, research groups have achieved superior specific capacitance using the prevalent hydrothermal and sol-gel techniques; nonetheless, our findings suggest that V_2_O_5_ nanorod produced through liquid-phase synthesis is expected to be superior to prior works. The synthesis of V_2_O_5_ nanorods, with an average length of 1 µm, was accomplished using the sol-gel route, as reported by Mumtaz et al. [24]. The resulting material demonstrated a specific capacitance of 365 F/g at 1 A/g, along with excellent energy and power characteristics of 12.67 Wh/kg at 247.5 W/kg. Mounasamy et al. [25] examined the electrochemical findings concerningV_2_O_5_/h-BN heterostructures synthesized via a hydrothermal process. In the composite, the V_2_O_5_ exhibited a 3D nanocube structure, which resulted in a better specific capacitance of 405 F/g. The capacitive contribution of the V_2_O_5/_h-BN material was determined to be 54% at 3 mV/s. The inclusion of h-BN suggests a potential competitive alternative to carbon hybrids.

We report on the electrochemical dynamics of V_2_O_5_ synthesized via a liquid-phase polyol method for supercapacitor applications. This strategy enables the formation of a native carbon coating directly on the V_2_O_5_ surface, eliminating the need for external carbon sources. A unique aspect of this work is the use of a series of glycols, ethylene glycol, diethylene glycol, triethylene glycol, and tetraethylene glycol, to facilitate the controlled nucleation and growth of monodispersed V_2_O_5_ nanorods. These glycols also play a dual role by contributing to the in situ formation of the carbon coatings, which significantly enhance the electrical conductivity of the material. Electrochemical evaluations of the glycol-derived V_2_O_5_ samples reveal significant improvements in the specific capacity, energy density, and power density, underscoring the effectiveness of this synthesis route for next-generation energy storage applications.

## 2. Materials and Methods

### 2.1. Materials

All the chemicals were of analytical grade and used as received. Ammonium metavanadate was purchased from NICE (London, UK). Ethylene, diethylene, triethylene and tetraethylene glycols were purchased from Alfa Aesar (Ward Hill, MA, USA) (extra pure).

### 2.2. Experimental Procedure

Monodispersed V_2_O_5_ was synthesized by liquid-phase synthesis by dissolving 0.5 g of ammonium metavanadate (NH_4_VO_3_) in 100 mL of ethylene glycol (EG) under magnetic stirring overnight to reach the homogeneous state of the solution. The solution was then shifted to a polyol setup with a temperature of 180 °C. A yellow cloud was detected to pile up gradually, darkening the entire colloidal solution. The turbidity of the solution remained almost constant. Refluxing was continued until particles had developed from the colloidal solution. The solution was stirred continuously for 2 h. The concentration of dispersed dark-blue-colored VO_2_ was slowly released from the stagnant solution when a state of critical supersaturation was reached. The dispersed colloidal nanoparticles were decanted with water and ethanol and then desiccated at 80 °C in a vacuum oven. To acquire the final product, V_2_O_5_, the glycolate precursors of VO_2_ were calcined in air at 500 °C for 3 h [26,27]. The aforementioned synthesis process was carried out by using diethylene glycol, triethylene glycol, and tetraethylene glycol separately. Specifically, the temperature for the reaction solution of diethylene glycol, triethylene glycol, as well as tetra ethylene glycol was maintained at 190 °C, 220 °C, and 278 °C, respectively.

### 2.3. Material Characterization

The powder X-ray diffraction pattern of the glycol-derived samples was characterized on the X-ray diffractometer of a PANalytical X’Pert Pro model with Cu Kα radiation (λ = 1.54 Å). The laser Raman spectra were measured using STR RAMAN, SEKI Corporation (Hachioji, Japan) to diagnose the vibrational properties. The thermogravimetric analysis of the uncalcined samples was performed using SDT Q600V20.9 (New Castle, DE, USA) in nitrogen surroundings. The valence states and the existence of the sample composition detected using X-ray photoelectron spectroscopy (XPS) were performedusing a PHI 5000 VersaProbe III. (Chigasaki, Japan). The energy-dispersive spectroscopic (EDS) patterns and morphology of the synthesized samples was scrutinized by field emission scanning electron microscopy (FE-SEM) andrecorded on a Quanta FEG 250 (Hillsboro, OR, USA). The typical TEM analysis of the synthesized samples was determined using a JEOL 2100+ (Tokyo, Japan).

### 2.4. Electrode Fabrication and Measurements

For the electrode preparation, an appropriate amount of active material, where the 80% ethylene, diethylene, triethylene and tetraethylene glycol-treated electrodes were designated as EV, DV, TV and TTV, respectively, 15% conductive carbon (Super P) and 5% binder polyvinylidene fluoride (PVdF) with a few accompanying drops of N-methyl-2-pyrrolidone (NMP) unvaryingly coated in nickel foam in the dimensions of (1 × 1 cm) were used. The electrodes were desiccated at 90 °C overnight in vacuum surrounding. The formulated electrodes EV, DV, TV and TTV, prepared by the aforementioned steps, were exploited as working electrodes. The mass of the active-material-coated nickel foam electrode was 2 mg. The three-electrode cell tests were conducted using a reference electrode (Ag/AgCl), a counter electrode (Pt), and the working electrode (EV, DV, TV and TTV). For the two-electrode cell, the anode was prepared by mixing Super P carbon and PVdF in a 1:1 ratio, while EV, DV, TV and TTV were exploited as cathode materials congregated with 3 M KOH electrolyte and infiltrate Whatman filter paper as a separator. For these measurements, cyclic voltammetry curves were measured in the voltage range from 0 to 0.6 V. The active materials were charged and discharged between 0 and 0.45 V and the current densities were varied according to the mass loading of the electrode materials. The electrochemical impedance spectroscopy was analyzed in the frequency interval between 100 kHz and 0.1 Hz. All the electrochemical measurements were evaluated using the OrigaLys Electrochemical workstation (Lyon, France).

The specific capacity (C_CV_, C_GCD_C/g) of the active materials was measured from the CV and GCD curves calculated using Equations (1) and (2) as follows:

(1) $$C_{\mathrm{CV}}\;=\frac{\int_{V_{0}}^{V_{1}}I\left(V\right)dV}{m^{\prime }v}$$

(2) $$C_{\mathrm{GCD}}=\frac{Idt}{m^{\prime }}$$

where $\;I\left(V\right)$ refers to the current (mA) values related to the specific potential (V) in the CV analysis, and $m^{\prime }$ and v denote the loading mass of the active materials (g) on the nickel foam substrateand the sweep rate of the CV curves (mV/s).

The charge storage mechanisms involved in the redox process of the glycol-derived samples were discriminated by the Trasatti method based on the following equations:

(3) $$q^{*}\left(v\right)=Constm^{\frac{-1}{2}}+{q^{*}}_{outer}$$

(4) $$q^{-1}\left(v\right)=Constm^{\frac{1}{2}}+{q^{-1}}_{total}$$

In Equations (3) and (4), q*(*v*) represents the capacity measured from the enclosed area of the CV curves, and q* and *q*^−1^ represent the capacity contributions related to the outer and total surface of the electrode. The variable ‘m’ corresponds to a function of the scan rate [28,29].

## 3. Results and Discussion

**Figure 1:**
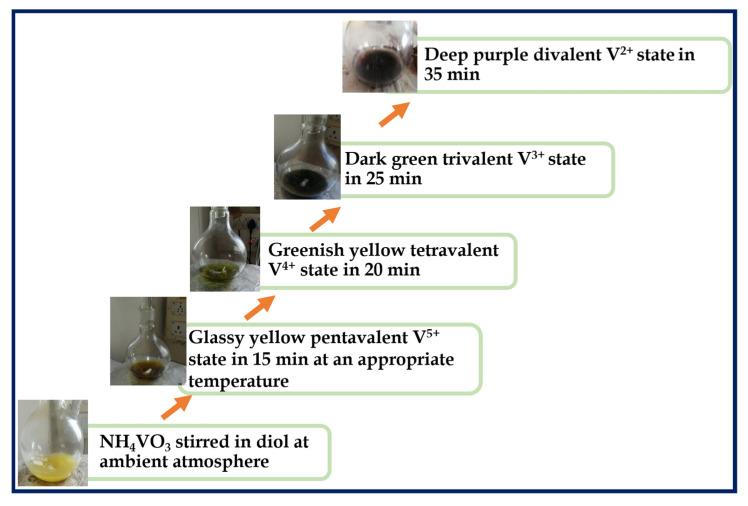
Schematic illustration of the reduction of NH_4_VO_3_ to V^2+^ compounds in ethylene, diethylene, triethylene and tetraethylene glycol.

Ammonium metavanadate could serve as a common vanadium pentoxide precursor source. In the one-pot liquid-phase synthesis of V^5+^, diols are oxidized to produce the corresponding V_2_O_5_ rods. The formation of monodispersed V_2_O_5_ by the thermal decomposition of NH_4_VO_3_ in diols and the reaction medium is represented schematically in Figure 1. The temperature was meticulously fixed at 180, 190, 220, and 278 °C for the respective solvents EG, DEG, TEG, and TTEG in the reaction medium, in accordance with the viscosity and nucleation rate of the solvent. The colloidal solution turned glassy yellow, lightened to green, then dark green, and finally, yielded a deep purple color product. The iridescence of ammonium metavanadate suggests that the reduction of the valence of vanadium species from V^5+^ to V^2+^ represents a convergence with all quartet glycol agents. The reaction seemed to be complete in a few process steps. The reduction rate was inferred from the time taken for the reaction mixture to change from pale yellow to deep green/blue, indicating the reduction of V^5+^ to V^2+^. In EG and DEG, this transition occurred within 30–45 min, whereas in TEG and TTEG, the reduction extended beyond 90–120 min (Figure S1). As a result of the visually apparent color changes, the colloidal solution reduced the NH_4_VO_3_ to the V^2+^ state within the temperature ranges of 180 °C, 190 °C, 220 °C and 278 °C. Determining theformation of V_2_O_5_ requires specific experimental parameters comprising time as well as temperature ranges. Carbon inevitably occupies the V_2_O_5_ lattice during polyol synthesis due to the organic medium of diols, the organometallic phase and the superficial oxidation of NH_4_VO_3_ with quartet glycols [30].

The plausible chemical reactions that tookplace in the reaction medium are represented by Equations (5)–(8) [31]. From the equations, initially, the NH_4_VO_3_ precursor affixedwith the glycols (EG, DEG, TEG and TTEG) to form the vanadyl glycolate precursor of the charge state (+2). Finally, the obtained glycolate precursor underwent annealing, transforming into V_2_O_5_.

The reaction between the precursor NH_4_VO_3_ and ethylene glycol can be expressed as

2NH_4_VO_3_ + C_2_H_6_O_2_ → 2VO(CH_2_O_2_) + 2NH_3_ + 2H_2_O (5)

In the case of diethylene glycol

2NH_4_VO_3_ + C_2_H_10_O_3_ → 2VO(CH_2_O_2_) + 2NH_3_ + 4H_2_O (6)

Triethylene glycol

6NH_4_VO_3_ + C_6_H_14_O_4_ → 6VO(CH_2_O_2_) + 6NH_3_ + 4H_2_O (7)

Tetraethylene glycol

8NH_4_VO_3_ + C_8_H_18_O_5_ → 8VO(CH_2_O_2_) + 8NH_3_ + 5H_2_O (8)

### Characterization of Diols-Derived V_2_O_5_

**Figure 2:**
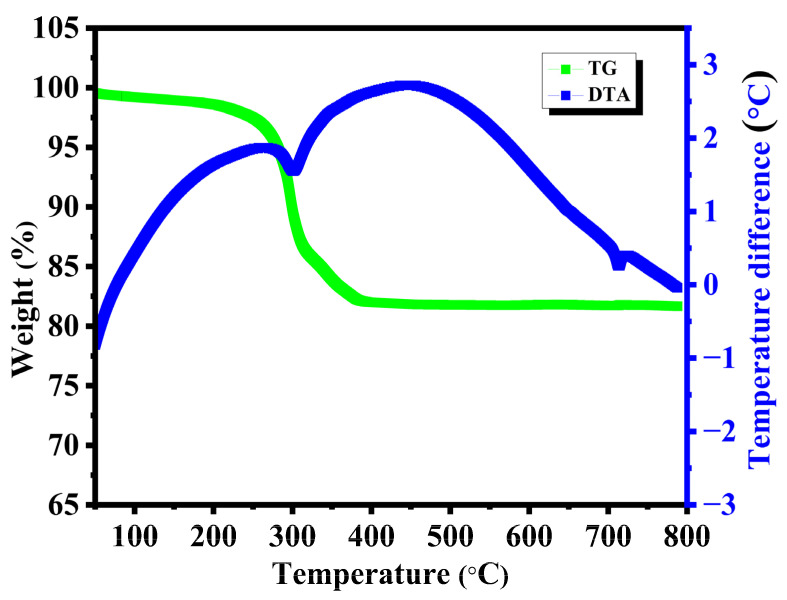
TGA-DTA curves of the vanadyl glycolate precursor in ethylene glycol solvent.

Figure 2 shows the TG and DTA results for the synthesized purple-colored precursor, prepared using ethylene glycol. During the heat treatment, the precursor decomposed in two stages to form V_2_O_5_ particles. From the TG curve, it is observed that the weight loss from room temperature to 250 °C indicates the removal of moisture, which was absorbed during the sample loading for the measurement. The second stage of weight loss, observed between 300 and 400 °C in the TG, is primarily due to the oxidation of vanadium from a divalent to a pentavalent state in the vanadyl glycolate precursor. It is reflected in the DTA curve with a small peak in between 270 °C and 340 °C. No significant thermal activity was observed above 400 °C in the TG curve, which is consistent with the formation of V_2_O_5_. The exothermic peak at 700 °C is the consequence of the reduced form of V_2_O_5_ [32,33]. The thermal analysis of the vanadyl glycolate precursor of EV demonstrates the probable temperature ranges for the DV, TV, and TTV samples. The oxidation of vanadium oxide to vanadium pentoxide can be expressed as

2VO_2_ + 1/2O_2_ → V_2_O_5_ (9)

**Figure 3:**
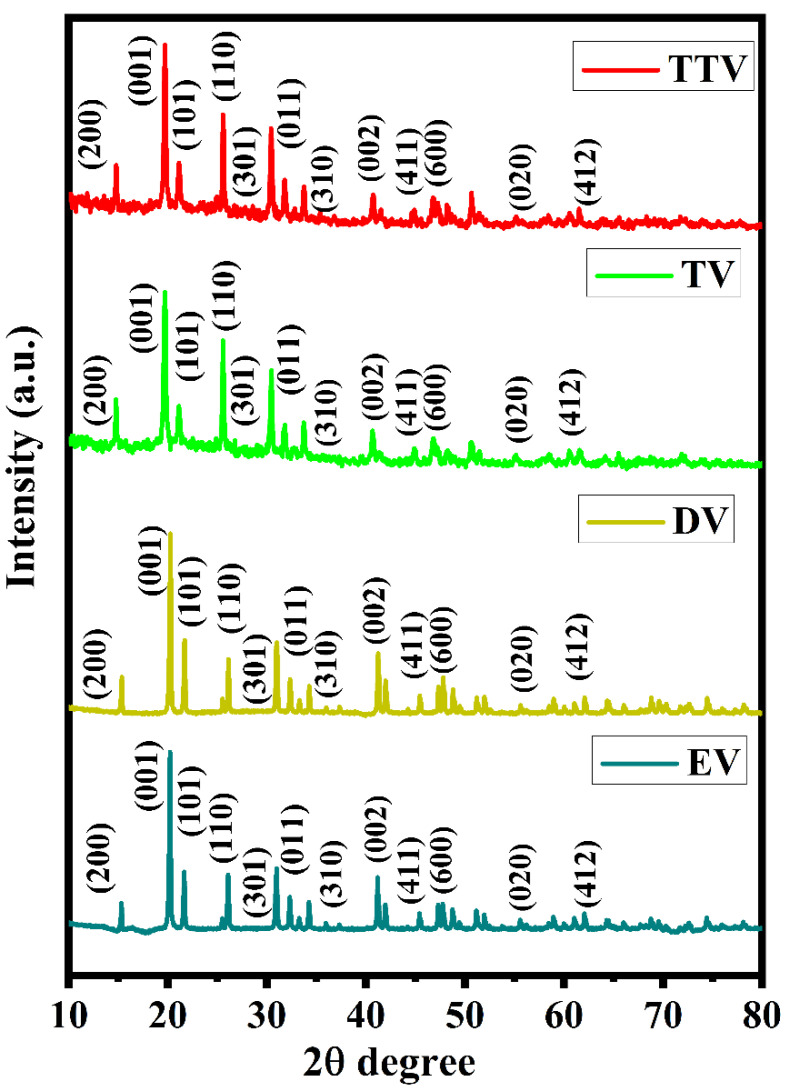
X-ray diffraction analysis of the V_2_O_5_ samples regarding the glycol representatives of EV, DV, TV and TTV.

The crystallinity of the V_2_O_5_ samples prepared using ethylene, diethylene, triethylene and tetraethylene glycols wasdemonstrated by XRD analysis. The X-ray diffraction patterns of EV, DV, TV and TTV are depicted in Figure 3. V_2_O_5_ demonstrates an orthorhombic layered phase, as indicated by the crystalline peaks that appeared at 2θ = 15.3, 20.26, 21.69, 26.13, 31.0, 32.35, 34.30, 41.29, 45.48, 47.36, 51.25, and 59.04°, which correspond to the (200), (001), (101), (201), (110), (301), (011), (310), (002), (411), (600), (020), and (412) planes, respectively. The crystallinity of the samples is attributed to the demonstration of monodispersed morphology for EV, DV, TV and TTV (JCPDS: 41–1426, space group: P*_mmn_*). The XRD patterns of the quartet samples show the core phase of V_2_O_5_ without any distinguishable peaks, which in turn revealsthe nonexistence of other VO*_x_*phases [34]. The crystallite size (D) of the prepared samples wasestimated using Scherrer’s formula

D = kλ/β cosθ(10)

In this equation, λ represents the X-ray wavelength (0.15406 nm), β is the full width at half maximum (FWHM), k is the shape factor constant (0.89), and θ is the Bragg’s angle.

(11) $$1/d_{\mathrm{ahl}}=\sqrt{\frac{h^{2}}{a^{2}}+\frac{k^{2}}{b^{2}}+\frac{l^{2}}{c^{2}}}$$

The lattice parameters were calculated using Equation (11) and the results are depicted in Table 1.

**Figure 4:**
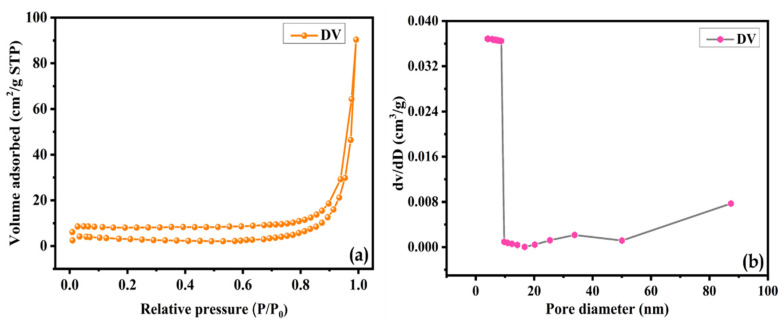
BET analysis of DV: (**a**) N_2_ adsorption–desorption isotherms and (**b**) BJH pore size distribution.

The porous characteristics of the DV sample were probed by nitrogen adsorption–desorption, as presented in Figure 4a. The material featured a type-IV isotherm with an H3 hysteresis loop at a high relative pressure range, possessing a specific surface area of 23.2 m^2^ g^−1^. A pore size of approximately 2.4 nm was determined from the BJH pore size distribution plot in Figure 4b, confirming its mesoporous nature. This characteristic is conducive to augmenting ion mobility and electron transfer, thereby endowing the sample with elevated electrochemical kinetics. Notably, the obtained specific surface area of DV is superior to other reported pristine V_2_O_5_ materials [35,36].

**Figure 5:**
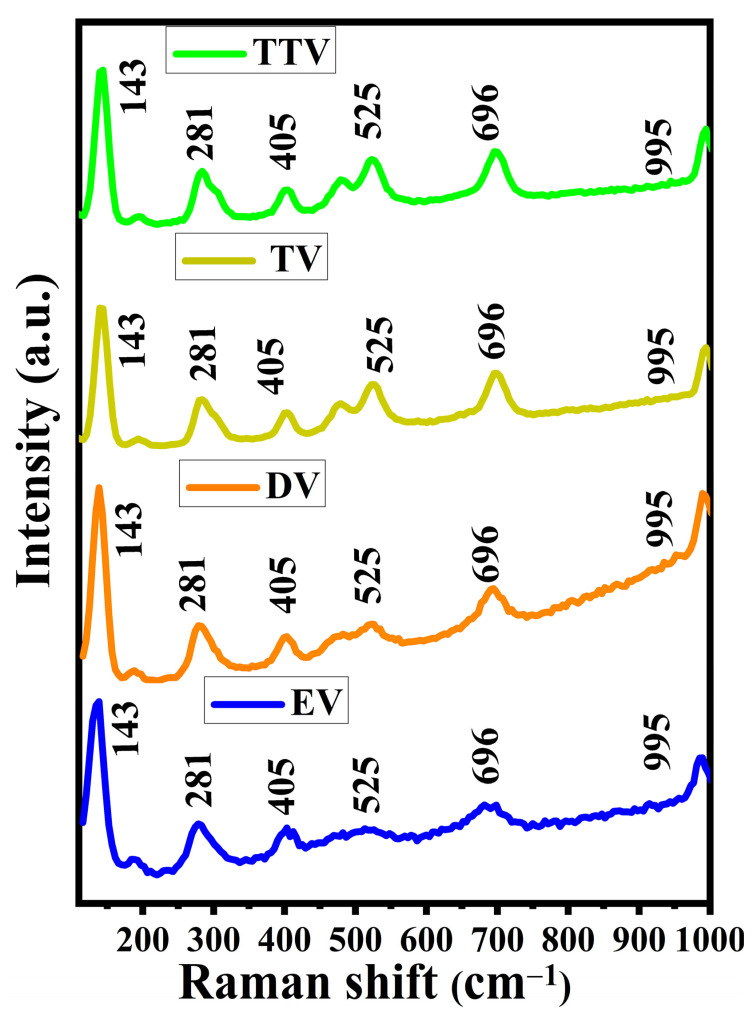
Raman spectra of the as-synthesized V_2_O_5_ calcined at 500 °C. The glycol-derived V_2_O_5_, EV, DV, TV and TTV are indicated in the blue, orange, green and black color, respectively.

Figure 5 shows the vibrational properties of the formed V_2_O_5_ material explored by Raman studies. The arising vibrational frequencies range from 100 to 1000 cm^−1^, ascribed to the orthorhombic phase of V_2_O_5_. The sharp dominant peak located at 144 cm^−1^ impliesan orderly dispersion of V_2_O_5_. The peaks at 144 cm^−1^, and 194 cm^−1^ provide a stretching vibration of the (V_2_O_2_)n mode of A_g_, B_3g,_ A_g,_and A_g_, respectively. The two bands around 281 cm^−1^ (B_2g_) and 405 cm^−1^ (A_g_) resemble a bending vibration of the O_3_–V=O and V–O_3_–V bonds. The peaks at 525 cm^−1^, 696 cm^−1^ and 995 cm^−1^ are attributed to the existence of the stretching mode of triply coordinated (V_3_–O), doubly coordinated (V_2_–O), and stretching vibration modes of the double bond (V=O), respectively [37]. In the enlarged plot (Figure S2), principally, the Raman spectra reveal that the reaction solution introduces surface-bound carbon residues onto the DV electrodes. The existence of a carbon is manifested by the peaks at 1742, 2781, 3486, and 3920 cm^−1^. The peak at 1742 cm^−1^ indicates the presence of C=O groups, which are characteristic of oxidized carbon, such as reduced graphene oxide (rGO). The peak at 2781 cm^−1^ corresponds to the D’ band (i.e., D-band in the second order), which is associated with defects in graphitic materials. Furthermore, the peaks at 3486 cm^−1^ and 3920 cm^−1^ represent O–H stretching modes in the prepared samples. It can be seen that the occupancy of carbon in the EV, TV and TTV samples is perhaps a minimal amount, which was not detected by the Raman analysis [38,39,40,41,42].

**Figure 6:**
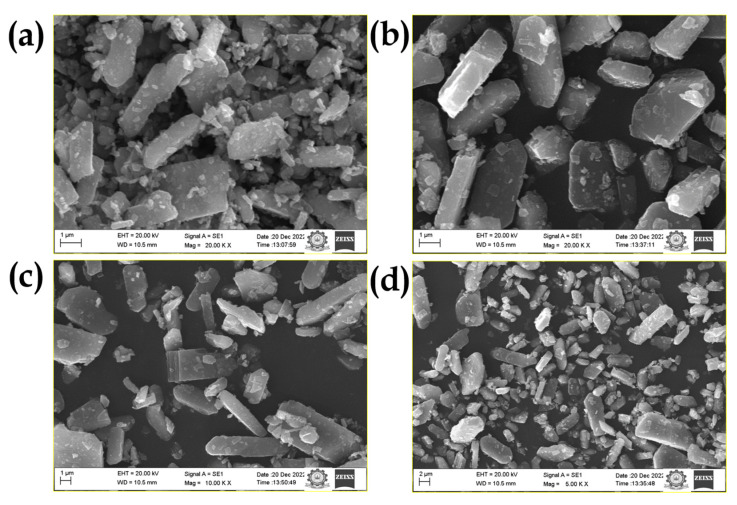
FE-SEM images showing the morphologies of V_2_O_5_ procured from (**a**) EV (**b**) DV (**c**) TV and (**d**) TTV.

The effect of ethylene, diethylene, triethylene and tetraethylene glycols on the surface morphology of V_2_O_5_ is shown in Figure 6a–d. The characteristic SEM images of the EV, DV, TV and TTV samples exhibit a prevalent dispersity of the rod morphology. The prepared V_2_O_5_ overtly exhibits a replicating morphology across the quartet solvents. Based on the morphology results, the hypothetical reaction process of NH_4_VO_3_ with glycol agents is as follows: (i) the vanadyl glycolate precursor is thermally decomposed to produce V_2_O_5_ rods, (ii) the controlled nucleation of vanadate ions from NH_4_VO_3_ fluctuates into various facets of rods with smooth edges, (iii) the parameters (temperature, reflux period) in the subsequent phase lead to the alignment of the vanadate ions to form a rod-like morphology, (iv) the viscous nature of glycols grants the additional significance of exempting V_2_O_5_ from agglomeration, and (v) the native V_2_O_5_ oxides are coated with a carbon layer, which originates from the organic media of the glycol agents. The morphology results of the glycol-derived samples are analogous to the preceding results published by Ragupathy et al. [26,43].

**Figure 7:**
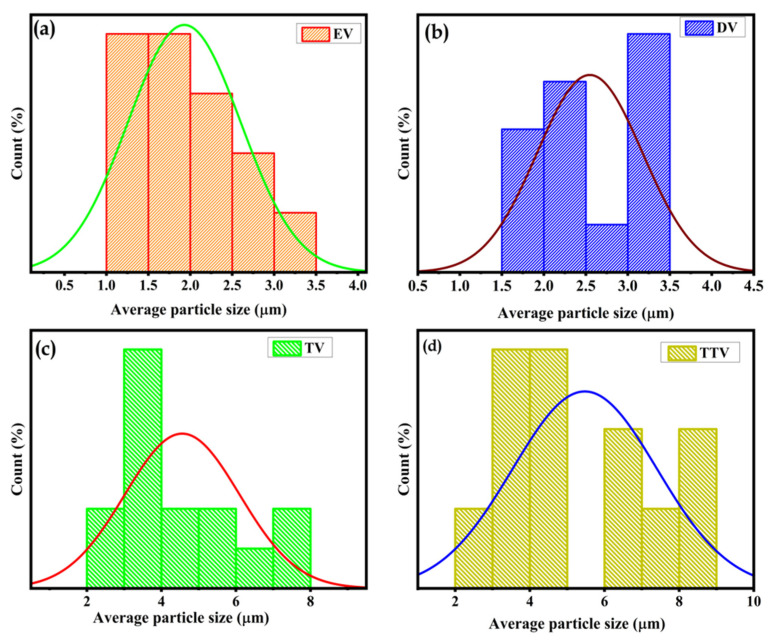
Particle size distribution of V_2_O_5_ in diverse glycols: (**a**) EV (**b**) DV (**c**) TV and (**d**) TTV, (green, purple, red and blue lines indicates the normal distribution curves).

The one-pot liquid-phase synthesis offers an intriguing particle size distribution for the synthesized samples. The estimated particle size distribution of EV and DV was found to be similar, ranging from 1 to 3.5 nm. Conversely, TV and TTV were determined to be analogous, ranging from 2 to 9 nm, as shown in Figure 7a–d. There was a conspicuous size variation among the samples, which suggested that the length of the glycols used during synthesis had a significant impact on the size of the nanoparticles.

The energy dispersive spectrum (EDS) was used to trace the metal composition of a prepared sample by using the characteristic X-ray energies. Vanadium, oxygen, and carbon were the main elements detected in the formed EV, DV, TV and TTV samples. The existence of V, O, and C elements was confirmed by the characteristic X-ray peaks that appeared in the spectrum. The EDS analysis disclosed the existence of carbon contents in the synthesized samples. In addition, the carbon present on the EV, DV, TV and TTV samples is revealed in Figure S3. The EV, TV, and TTV samples exhibited a lower carbon content relative to DV, and this was considered to be a factor contributing to the enhanced charge storage behavior of the DV sample.

**Figure 8:**
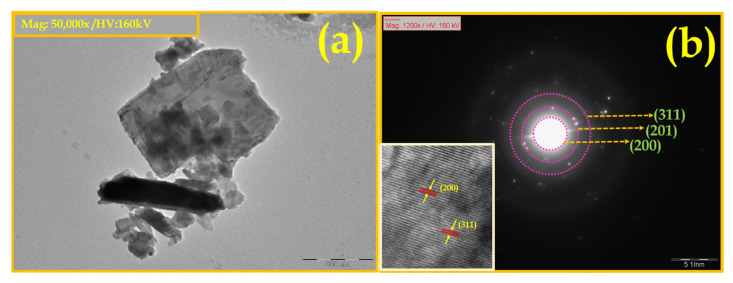
TEM image of (**a**) DV and (**b**) SAED pattern and inset represents the d-spacing of the DV electrode.

The magnified TEM image of the DV sample presented in Figure 8a confirm the rod morphology. The infusion of carbon into the sample was undetectable, likely because the carbon existed as oxygenated species. The exposed carbon on the DV sample was considered to enhance the electrochemical reaction sites [44]. The selected area diffraction pattern of DV revealed a well-defined corona of discernible spots, which matched up with the corresponding (200), (201) and (311) planes of V_2_O_5_ and disclosed the polycrystalline characteristics of the sample in Figure 8b.

**Figure 9:**
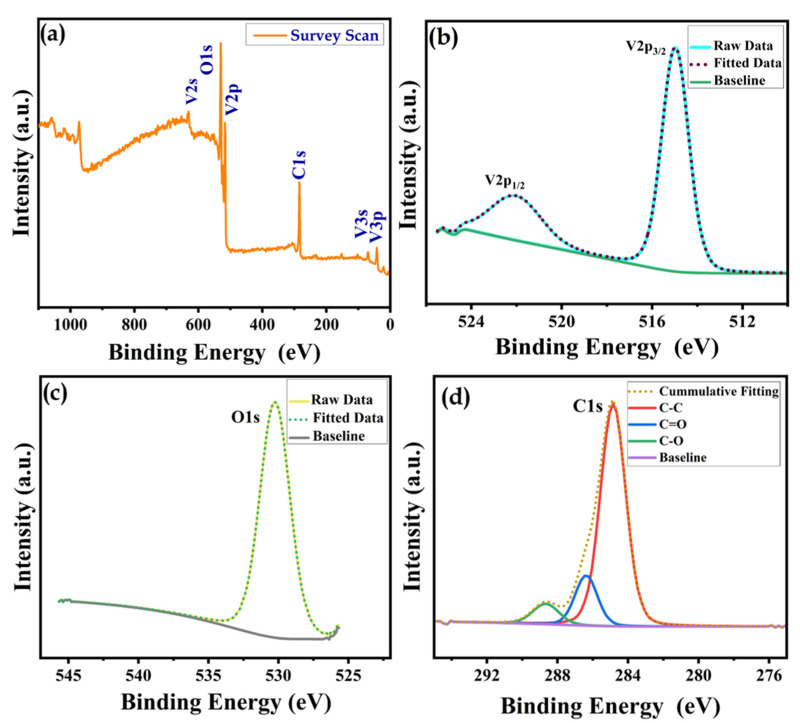
XPS spectra of the (**a**) survey scan of DV, (**b**) V2p, (**c**) O1s and (**d**) C1s.

From the perception of the charge storage performance across the variant solvent samples, DV attained the highest specific capacity values and enhanced stability properties. Hence, the surface composition and chemical state of the elements in the DV sample were characterized by XPS. Figure 9a shows the survey spectrum encompassing V, O and C, which is probably well-coordinated with the EDS analysis of DV. In the XPS, the binding energy peaks indicate the appropriate presence of vanadium in the V^5+^ valence state. The characteristic core-level V^5+^ spectra displayed two distinct peaks at the binding energies of 517 eV and 527 eV, indexed to the spin states of 2p_3/2_ and 2p_1/2_, pointing out the formation of V_2_O_5_ in the polyol solvents, as depictedin Figure 9b. From Figure 9c, the O1s positioned a peak at a binding energy of 530.5 eV, which corroborates the bonding of oxygen with vanadium ions [45]. The characteristic C1s spectrum was deconvoluted into three peaks at 285.4 eV, 286.9 eV and 288 eV, which were ascribed to the chemical bonds of C–C, C–O and C=O, respectively, as shown in Figure 9d.

**Figure 10:**
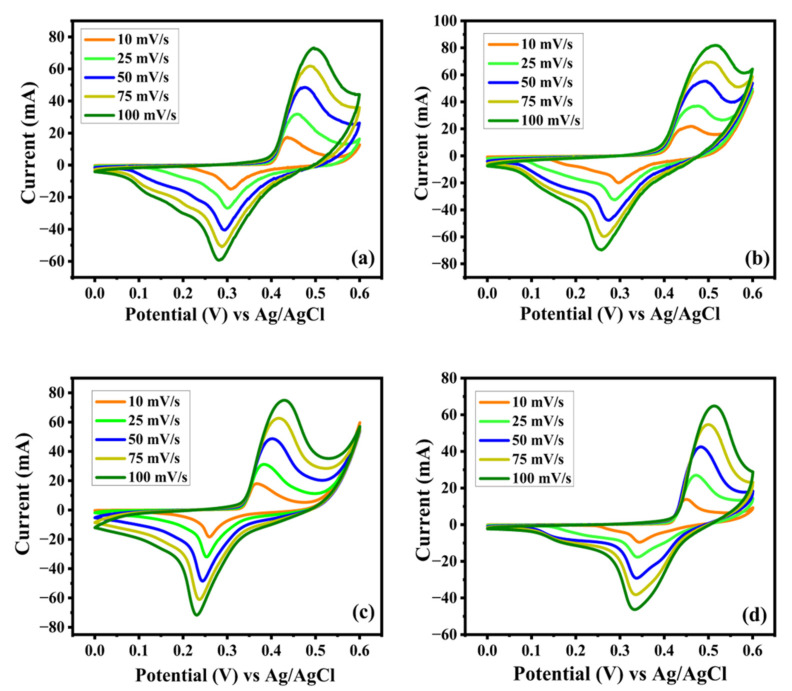
(**a**–**d**) Cyclic voltammetry (CV) curves of the (**a**) EV (**b**) DV (**c**) TV and (**d**) TTV electrode at divergent scan rates ranging from 10 to 100 mV/s.

The techniques of cyclic voltammetry, galvanostatic charge–discharge, and electrochemical impedance spectroscopy (EIS), which aimed to determine the electrochemical properties of the synthesized electroactive samples, were tested using the three-electrode system detailed in Figure 9a–d and Figure 10a–d. The comparative CV characteristics of EV, DV, TV and TTV were measured at various scan rates in the potential interval of (0–0.6 V), revealing a strong couple of redox peaks without a rectangular pattern, corroborating that the host material (V_2_O_5_) consumes charges via the intercalative (battery-type) mechanism. From Figure 10a–d, the electrodes have shown a CV pattern for EV, DV, TV and TTV that indicates the sequential redox reaction of V^5+^ ⇌ V^4+^. The shapes of the voltammetry curves of the synthesized electrodes are slightly different from each other.

**Figure 11:**
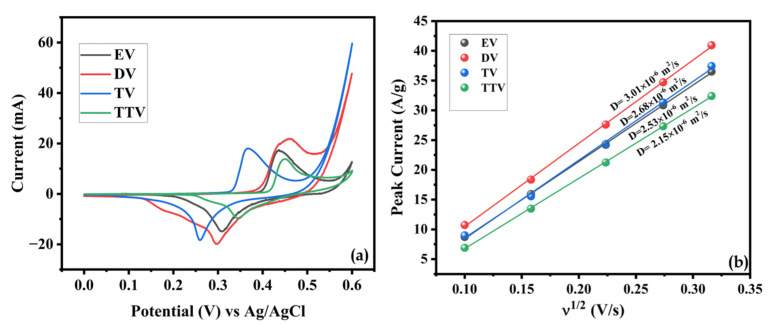
(**a**) CV response of the prepared samples at a fixed scanning speed of 10 mV/s and (**b**) the linear dependence of $i_{p}onv^{\frac{1}{2}}$ highlights the differences in diffusion coefficients among the synthesized samples.

The deviance of the CV curve is elevated with the specific capacity of the prepared samples in the following order: TTV < EV < TV < DV. Upon obtaining the CV results, it can be observed that the DV electrode presents the superior specific capacity of 453.1 F/g at 10 mV/s. The remaining EV, TV, and TTV attain a lower specific capacity than DV, ranging from 184.9 to 114.3 C/g at the same scan rate, respectively (Figure S4a). It is worthy of attention that the increased percentage of carbon coating expedited the sluggish redox kinetics, making the DV electrode extremely active toward charge storage and hinting at its contribution to the superior specific capacity values. The potential was increased during oxidation and the potential was decreased during the reduction process upon increasing the scan rate along with the increment in the integral area of the CV curves. Figure 11a illustrates the comparison of the CV curves of the prepared samples at 10 mV/s. The DV sample shows a larger integral area than the other samples studied at the same scan rate. This indicates the superior current-to-potential response of DV and demonstrates enhanced redox kinetics and reversibility. The diffusion coefficients of the prepared samples were deduced from the Randles–Sevcik equation, which is given below.

(12) $$i_{p}=\left(2.69\times 10^{5}\right)n^{\frac{3}{2}}AD^{\frac{1}{2}}Cv^{\frac{1}{2}}$$

where $i_{p}$ is the peak current (A), n is the number of electrons transferred (approximately *n* = 1), A is the contact area between the V_2_O_5_ electrode and the KOH electrolyte, C is the electrolyte concentration (approximately 3 mol/cm^3^), D is the diffusion parameter (cm^2^/s) and v is the scan rate. Based on this equation and the linear fitting of $i_{p}$ versus $\;v^{\frac{1}{2}}$, the calculated diffusion coefficients are depicted in Figure 11b. The DV electrode exhibits a higher diffusion capability and superior reversibility compared to the EV, TV, and TTV electrodes.

**Figure 12:**
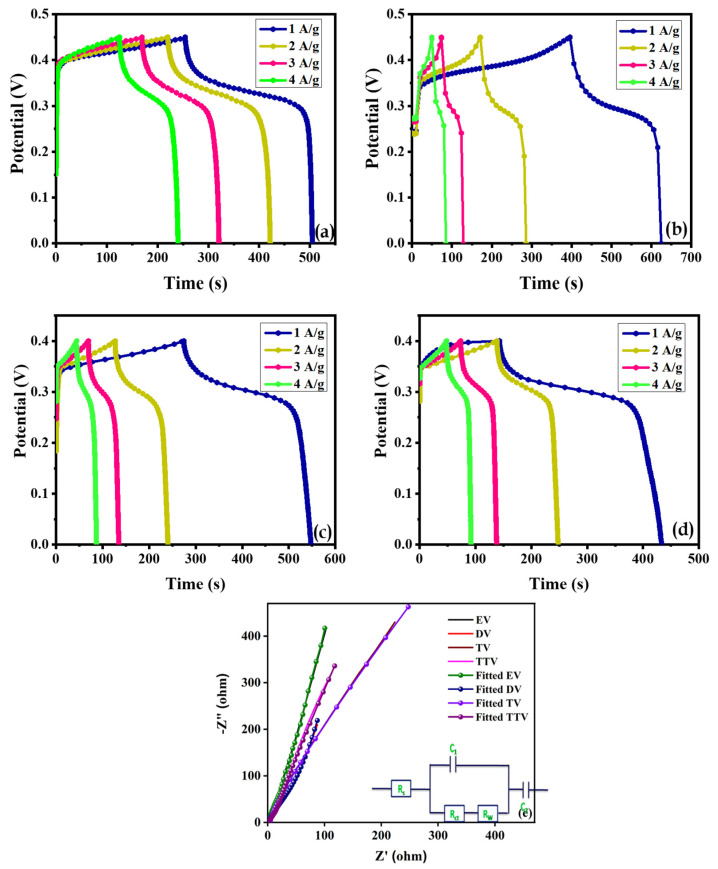
(**a**–**d**) Charge discharge curves of EV, DV, TV and TTV electrodes, and (**e**) Nyquist plots of the synthesized electrodes.

Figure 12a–d illustrate the charge–discharge characteristics of the quartet of glycol-derived EV, DV, TV and TTV samples with altering current densities (1–4 A/g). The samples reveal non-linear charge–discharge plateaus with typical battery-type behavior. The DV sample exhibits the longest discharge time, which accounts for the higher quantity of carbon existing on the electrode, enough to provide better electrochemical activity than the EV, TV and TTV samples. Significantly, the smaller IR drop values indicate the finer conducting nature of the electrodes. As expected, the DV electrode performance is enhanced compared to other electrodes. The optimistic specific capacity values of the quartet of glycol-derived battery-type V_2_O_5_ electrodes compared with the quintessential current densities are displayed in the plot (Figure S4b). The specific capacity values calculated for EV, DV, TV and TTV were 275.3, 460.0, 286.3 and 225.6 at 1 A/g. The specific capacity values calculated from the GCD analysis were comparable, which was impacted by the discharging rates of the battery-type V_2_O_5_ electrode material. The electrochemical performance of the prepared DV sample has been compared with previously published reports, as listed in Table 2.

EIS measurements were used to probe the electronic conductivity of the electrode materials. Figure 12e shows the electron transfer kinetics of the electrodes that were analyzed in the frequency range of 100 kHz to 0.01 Hz. The initial intercept on the *x*-axis indicates effective series resistance (R_s_), the semicircle in the high-frequency region estimates the charge transfer resistance (R_ct_)and the capacitive process is demonstrated by an oblique line in the low-frequency region.

The Nyquist plots of all the synthesized EV, DV, TV and TTV show relatively oblique lines at low frequencies, signifying the exceptional diffusion-controlled capacitive behavior of the electrodes. That there is no semicircle coming into view in the high-frequency region represents a lesser amount of (R_ct_) and promotes fast ion diffusion/transfer during faradic reactions [17]. The fitted equivalent circuit (inset) and the extracted parameters, such as the solution resistance (R_s_), charge transfer resistance (R_ct_), and Warburg impedance (W_d_), are listed in Table 3.

**Figure 13:**
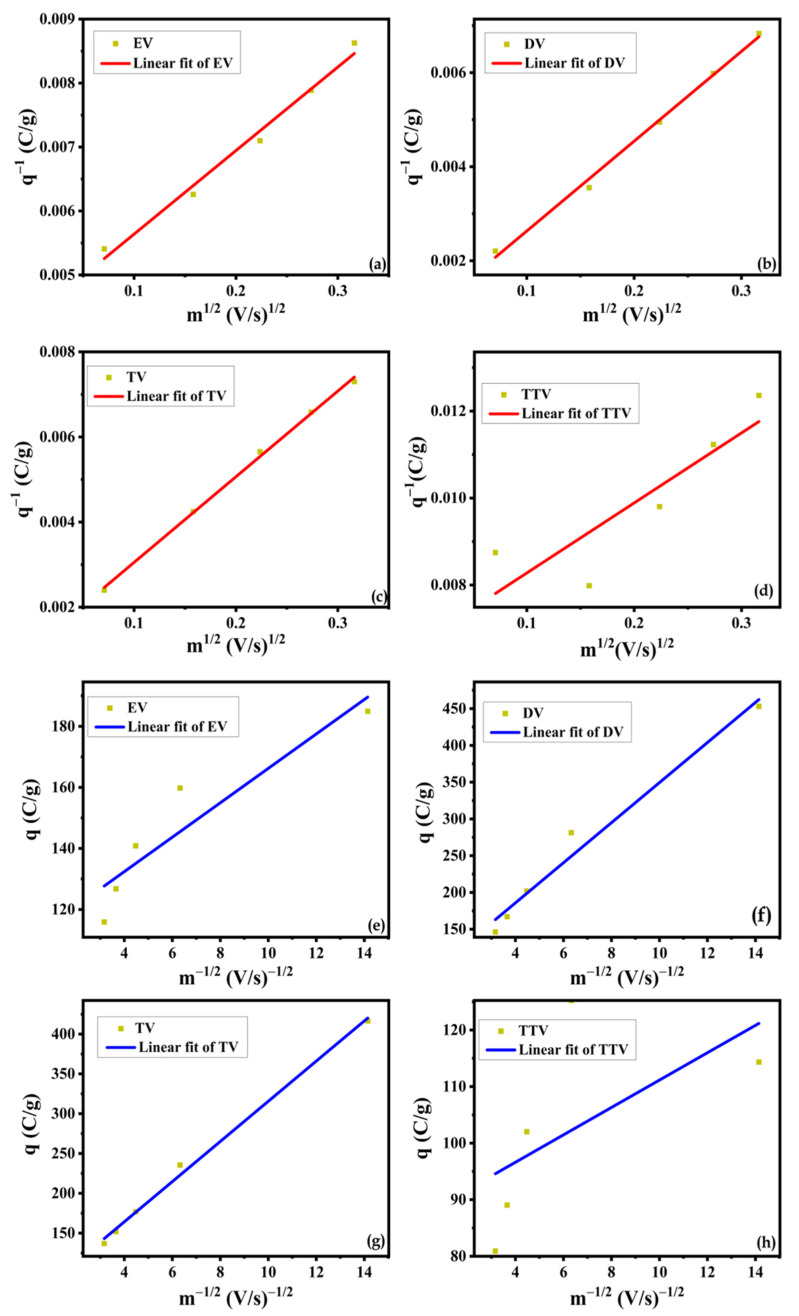
Trasatti plot of the EV, DV, TV and TTV samples: (**a**–**d**) m^1/2^ vs. q^−1^ and (**e**–**h**) m^−1/2^ vs. q.

**Figure 14:**
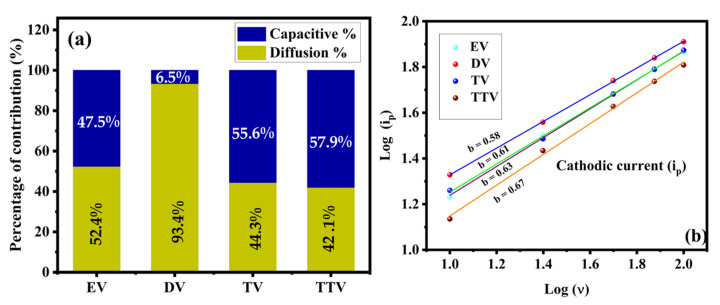
(**a**) The contribution of the diffusion-controlled capacity and non-diffusion-controlled capacity of the synthesized electrode materials and (**b**) the relationship between the peak currents’ variation with the respective sweep rates (b value) determined for the glycol-derived samples.

For the respective electrode materials, the Trasatti method relies on evaluating the distinct contributions of surface-controlled (outer capacitance) and diffusion-controlled (inner capacitance) processes in complete redox kinetics. On account of the charge storage mechanisms, the total capacitance (*q*^−1^) is measured as the sum of the inner (*q*^#^) and outer surface capacitance (q*) of the electrode reaction at different scan rates. From the plots of q* vs. m^−1/2^ and *q*^−1^ vs. m^1/2^, the linear regions’ intercept value estimates the capacitance stored at the outer surface (q*) and total surface (*q*^−1^) of the electrode by extrapolating the scan rates to infinity and zero. The capacitance of the inner surface (*q*^#^) is determined from the difference between *q*^−1^ and q*. Figure 13a–f present the Trasatti plots of the electrode materials. The estimated outer capacitance values as a percentage of the total capacitance of EV, DV, TV and TTV are found to be 109.8, 63.3, 76.8 and 86.9 Cg^−1^. Comparably, for the inner capacitance, the calculated values are 121.0, 898.1, 61.3 and 62.9 Cg^−1^, respectively. The ratio of *q*^#^/*q*^−1^ and q*/*q*^−1^ explored by the Dunn method provides the percentage contribution of EV, DV, TV and TTV, as displayed in Figure 14a.

Notably, the surface-controlled process was dominant in the EV, TV and TTV electrodes, suggesting that the electrochemically active sites were mostly assessed by the surface-controlled process rather than the DV electrode [51].

To further elucidate the accumulated capacitive and non-capacitive effects on the total charge storage, they can also be extracted through the equation describing the relation between the CV currents under various sweep rates expressed as

(13) $$i_{p}={av}^{b}$$

Here, $i_{p}$ and v represent the measured CV current and scan rate at a fixed potential range, while a and b are adjustable parameters found from the slope value of the linear fitting between log ($i_{p}$) and log (v). It is commonly known that a capacitance rise is imputed by the diffusion-controlled (*b* = 0.5) and surface-controlled/pseudocapacitive process (*b* = 1). For our report, the obtained b values are plotted in Figure 14b. From the plot, it is conclusive that the DV electrode (*b* = 0.58) stored charge primarily by a diffusion-controlled rather than a capacitive process. Quite significantly, the b value for the EV, TV and TTV electrodes falls between 0.5 and 1; hence, the electrodes exhibita combined storage contribution of capacitive and non-capacitive processes. From this, the b value emphasizes the capability of detecting the charge storage mechanisms of electrode materials.

The total current at 10 mV/s with a fixed potential limit of 0.6 V can be derived from the equation

(14) $$i\left(v\right)=k_{1}v+k_{2}v^{\frac{1}{2}}$$

where $i\left(v\right)$ stands for the measured current (A), v signifies a scan rate (mV/s), the first and second terms denote the surface-controlled currents and diffusion-limited currents, and $k_{1}$ and $k_{2}$ are appropriate values. The values of $k_{1}$ and $k_{2}$ are defined as the slope and intercept derived from the plot between $v^{\frac{1}{2}}$ and $\frac{i}{v^{\frac{1}{2}}}$. This result implies that the CV curves in Figure S5a–d show the diffusion contribution and surface-controlled contribution for the EV, DV, TV and TTV samples. Considering the results, at diminutive scan rates, the electroactive sites of the samples predominantly store charges by a diffusion-controlled process and minimize the surface-controlled contribution. It was observed that all four samples seemed to be similar in the diffusion contribution to the total capacitance. The computed diffusion-controlled contributions of EV, DV, TV and TTV were found to be 83.8%, 90.8%, 83.8% and 80.2% and the samples held 16.1%, 9.1%, 16.1% and 19.7% of the surface-controlled contribution, respectively [52].

**Figure 15:**
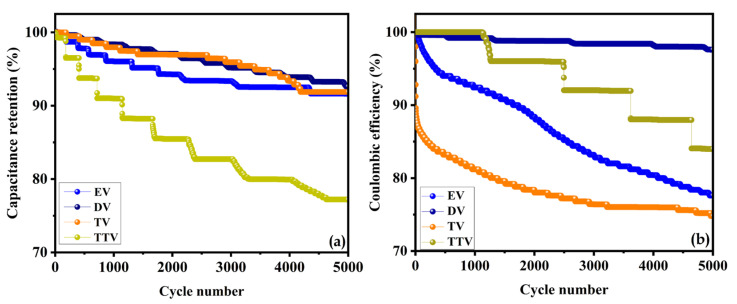
Comparative analysis of the (**a**) electrochemical stability and (**b**) coulombic efficiency of the synthesized samples using ethylene, diethylene, triethylene and tetraethylene glycols.

The electrochemical stability of electrode material is a prominent factor in supercapacitor application. The cyclic stability of EV, DV, TV and TTV was analyzed in a consecutive charge–discharge cycle over 5000 cycles at a current density of 4 A/g, as illustrated in Figure 15a. The capacities of the EV, DV, TV and TTV electrodes were retained at 87.2%, 94%, 91.4% and 83.7%, respectively. The electrodes sustain the same pace of cycles and their performance extends up to the final cycles due to the better diffusion rate of the redox process (Figure S6). The DV sample exhibits the highest coulombic efficiency, accompanied by a slight capacity decrease of 2.4% (Figure 15b). The other samples maintain reasonable coulombic efficiency values of 78.2%, 77.6%, and 83.9% for EV, TV, and TTV, respectively.

**Figure 16:**
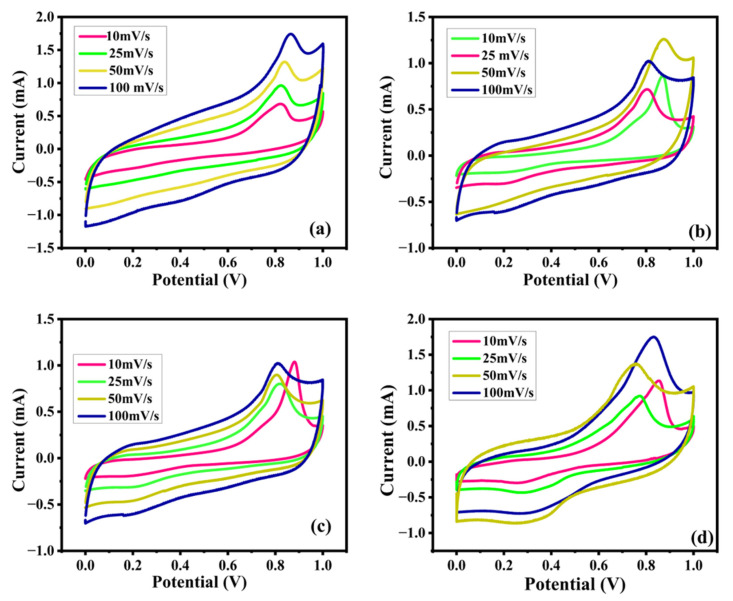
(**a**–**d**) Cyclic voltammetry (CV) scanned between 0 and 1.0 Vat various sweep rates: (**a**) EV//AC (**b**) DV//AC (**c**) TV//AC and (**d**) TTV//AC asymmetric devices.

Device application is the decisive measure of the prepared electrodes in a two-electrode configuration. The assembled hybrid device employs a battery-type EV, DV, TV and TTV as a positive electrode, where carbon black functions as the negative electrode material. The CV curves of the hybrid device were recorded in a widened voltage interval of 0–1 V. The symmetrical CV curves of the hybrid devices in Figure 16a–d exhibit the faradic charge stowage peaks instigated by the V_2_O_5_-positive electrode material. The hybrid device did not exhibit the EDLC characteristics of the carbon black anode material. The mass ratio of the anode and cathode was close to 2 mg.

**Figure 17:**
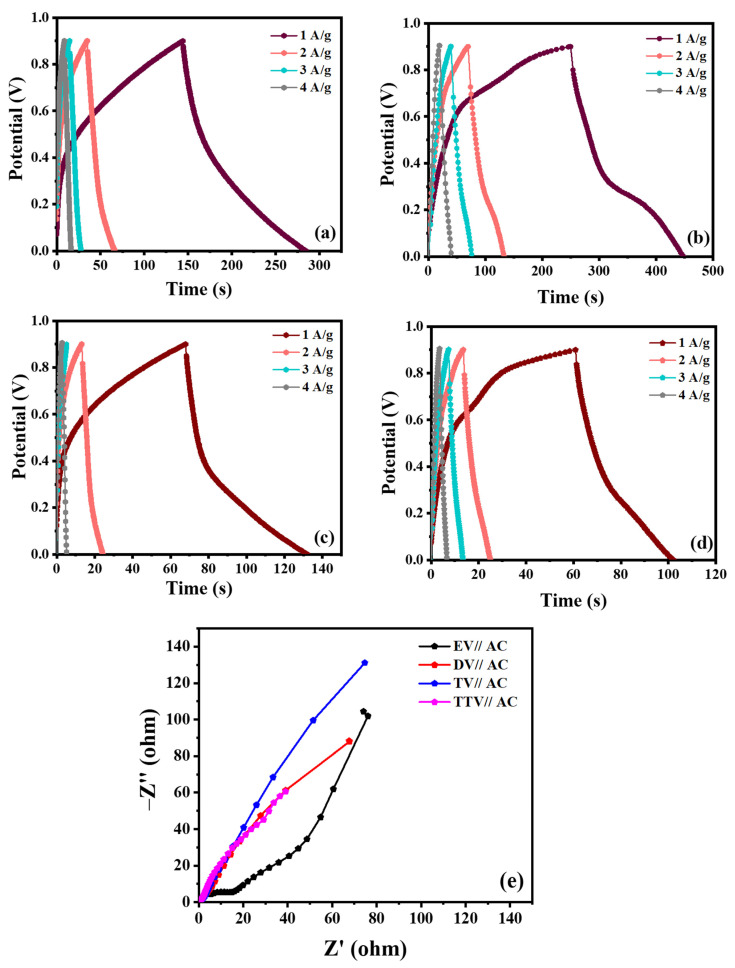
(**a**–**d**) Charge–discharge profiles of the fabricated asymmetric devices at a range of current densities (1–4 A/g): (**a**) EV//AC (**b**) DV//AC (**c**) TV//AC (**d**) TTV//AC and (**e**) Nyquist plots of the fabricated devices.

Figure 17a–d illustrate the typical non-linear response and disclose a low IR drop for the four hybrid devices, indicating the supreme exploitation of the V_2_O_5_ cathode material. In the meantime, the DV//AC hybrid device demonstrates a marginally higher capacity than EV//AC, TV//AC and TTV//AC, respectively. The specific capacity of the full cell was found to be 92.8, 131.4, 42.4 and 27.2 for the EV//AC, DV//AC, TV//AC and TTV//AC devices at 1 A/g.

The EIS measurements revealed the response of the hybrid devices (Figure 17e). All four hybrid devices (EV//AC, DV//AC, TV//AC and TTV//AC) exhibit a depressed semicircle and a slanting line in the high and low frequency ranges. The initial point on the *x*-axis represents the solution resistance (R_s_), which relates to the electrolytic resistance. The depressed semicircle refers to the charge transport resistance (R_ct_), and theslanting line denotes the diffusion.

The energy density (E_d_) and power density (P_d_) were extracted from the GCD curves of the hybrid devices using the following equations:

(15) $$E\;=\;\frac{1}{2}C\;{\left(\Delta V\right)}^{2}$$

(16) $$P=\frac{E}{\Delta t}$$

 where ‘C’ is the specific capacitance, and Δ*t* (s) denotes the discharge time within the potential range ΔV, respectively.

**Figure 18:**
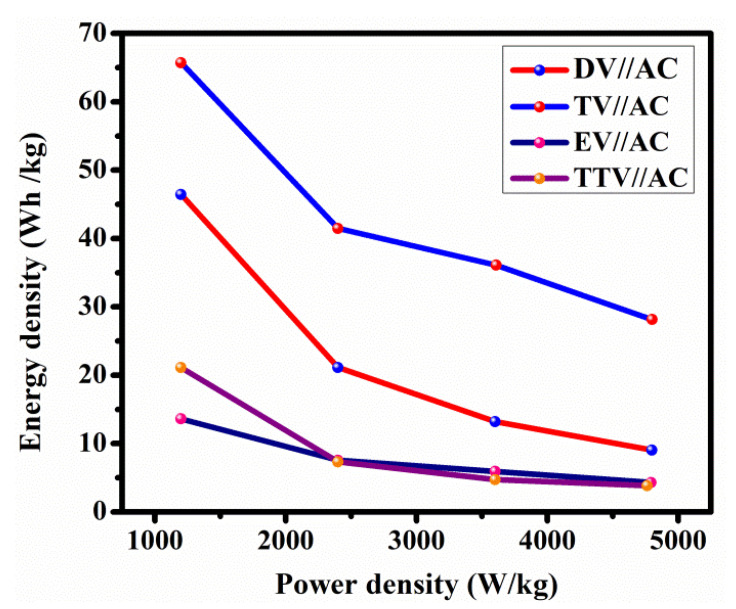
Ragone plots of the fabricated asymmetric devices EV//AC, DV//AC, TV//AC and TTV//AC.

The operating features of the hybrid device confer the energy and power densities (Ragone plot) resulting from the charge–discharge currents, which are pictorially represented in Figure 18. The constructed DV//AC device achieves a maximum energy density of 65.7 W h/kg at a power density of 1199.9 W/kg, while the energy and power densities of EV//AC, TV//AC and TTV//AC were foundto be 46.4 W h/kg at 1199.9 W/kg, 21.1 W h/kg at 1194.3 W/kg, and 13.6 W h/kg at 1199.8 W/kg.

The structural and morphological characteristics of the DV electrode before and after cycling were meticulously examined to elucidate the changes resulting from prolonged electrochemical assessment for 2000 cycles, which showed a capacitive retention of 93.5% (Figure S7). The XRD patterns of the coated (pre-cycling) and cycled (post-cycling) DV electrodes in Figure S8a exhibited distinct V_2_O_5_ reflections (marked by *), confirming the retention of the active material’s crystal structure even after 2000 charge–discharge cycles. However, a slight reduction in the peak intensities was observed in the cycled sample, suggesting minor degradation or partial amorphization of the active material upon extended cycling (Figure S8b, see enlarged view).

SEM analysis further supports the observations illustrated in Figure S9a–c. Prior to cycling, the DV material uniformly coats the nickel foam, effectively covering its surface roughness and protuberances, which confirms successful electrode fabrication. After cycling, the SEM images of the electrolyte-parched electrodes still reveal the presence of DV material adhered to the substrate (highlighted by dotted lines), indicating good morphological stability (Figure S10a–c). These results collectively confirm that the DV electrode sustains both structural and morphological integrity in an asymmetric device configuration, even after extended cycling.

These results demonstrate superior energy density values compared to previously reported V_2_O_5_ electrode material [18,53,54,55,56], as presented in Figure S11. The consolidated insights into the electrochemical performance of the quartet of glycol-derived battery-type V_2_O_5_ electrode materials suggest their potential as appropriate materials and ensure their potent use in supercapacitor applications. However, scrutinization of the V_2_O_5_ electrode materials synthesized in different polyols demonstrates various faradic kinetics.

**Figure 19:**
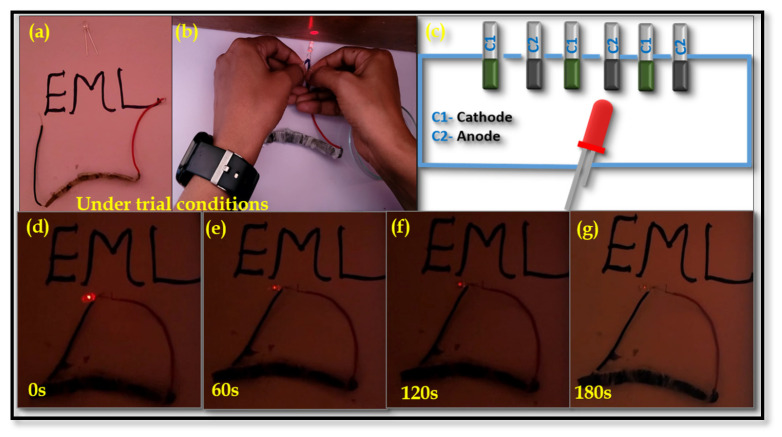
Digital photographs showcasingthe practical demonstration of an asymmetric DV//AC device comprised of three cells in a series connection. (**a**,**b**) Under trial conditions and the light response of the DV//AC device. (**c**) The schematic representation of the series connection and (**d**–**g**) show the asymmetric device lighting a red LED at different intervals.

Based on the practical manifestation, the active materials DV (cathode) and AC (anode) were separately coated on three pairs of nickel foam with dimensions of 2 × 1 cm^2^ and assembled in a series connection. The fabricated device was charged for 30 s to power an LED. After disconnecting the charging source, the light-responsive behavior of the device was recorded, as shown in Figure 19a–g. The LED remained lit for 180 s. Initially, the LED displayed high brightness (initial level), followed by a slight decrease in intensity after 180 s. This result suggests that the practical demonstration supports the successful application of the prepared electrodes in supercapacitor devices.

Taking all the above outcomes into account, concretely, the following features govern the reaction solution and contribute to the valency of V^5+^ monodisperse rods. (1) No effort has been made to control the particle growth, while the nucleation and growth rate of the particles are restrained by glycols. (2) The polyol solvents displays multifunctional properties of reducing, dissolving and stabilizing agents in theliquid-phase synthesis of colloidal nanoparticles. (3) The generation of particle formation in a colloidal solution is indicated by a color transition visible to the naked eye. (4) Agglomeration is negated by acquiring a monodispersity of V^5+^ rods. (5) Significantly, the carbon coating generated during the synthesis process merges with the active materials and facilitates better electrochemical kinetics for superior specific capacity values. (6) The reduction rate of NH_4_VO_3_ is quickly achieved in ethylene and diethylene glycols, in contrast to triethylene and tetraethylene glycols.

Seamlessly, DV outperforms the other glycols (EV, TV, and TTV) due to its precise molecular structure and thermal decomposition properties. The chelation characteristics of DV enable it to effectively bind with metal precursors and promote a higher degree of carbon intermediate generation. While TV and TTV offer a favorable carbon-to-oxygen ratio, the steric hindrance arising from their larger structures limits the formation of carbon species. Conversely, EV, with its lower carbon-to-oxygen ratio, produces fewer carbon species compared to diethylene glycol. The trend of carbon deposition follows this sequence: DV > EV > TV > TTV.

## 4. Conclusions

Our investigation successfully synthesized V_2_O_5_ nanorods using various glycols (ethylene, diethylene, triethylene and tetraethylene). The choice of glycol was found to significantly influence the material’s morphology and faradic charge storage capabilities. The diethylene-glycol-derived V_2_O_5_ (DV) electrode exhibited superior performance, delivering a high specific capacity of 453.16 C/g at 10 mV/s and 460.2 C/g at 1 A/g. It also demonstrated exceptional prolonged stability, with 94% of its capacitance preserved after 5000 cycles. When integrated into an asymmetric supercapacitor, the DV electrode yielded a high energy density of 65.7 Wh/kg, which positioned this synthesis method as a viable route for developing next-generation supercapacitors.

## Figures and Tables

**Table 1:** Structural parameters of the prepared samples.

Sample Codes	Lattice Parameters (Å)			Crystallite Size (nm)
	*a*	*b*	*c*	
EV	11.50810	4.37324	3.56388	64.50
DV	11.51290	4.37184	3.55953	63.23
TV	11.50952	4.37942	3.56324	64.37
TTV	11.52141	4.36976	3.55246	65.51

**Table 2:** Comparison of the electrochemical properties of the DV electrode with other reported V_2_O_5_-based materials.

Samples	Methods	Morphology	Specific Capacitance (F/g)	Current Density (A/g)	Electrolytes	Ref.
rGO@V_2_O_5_	Solvothermal	Nanorods	450.5	0.5	5 M LiNO_3_	[46]
V_2_O_5_ NRs	Greensynthesis	Nanorods	149.1	1	2 M KOH	[47]
VO*x*@C	Sol–hydrothermal	Nanorods	548	0.5	1 M Na_2_SO_4_	[48]
V_2_O_5_	Hydrothermal	Nanorods	347	1	1 M LiClO_4_	[49]
Sn-V_2_O_5_/rGO	Sol-gel	Nanorods	159.3	1	1 M Na_2_SO_4_	[50]
V_2_O_5_	Sol-gel	Nanorods	365	1	2 M NaOH	[24]
DV	Liquid-phase synthesis	Nanorods	460	1	3 M KOH	This work

**Table 3:** The extracted parameters from the equivalent circuit for the synthesized samples.

Sample Code	R_s_ (Ω)	R_ct_ (Ω)	Z_w_ (Ω)
EV	0.9	0.4	1.55
DV	0.53	0.2	4.24
TV	3.8	0.27	1.78
TTV	1.11	0.45	1.32

## Supplementary Materials

The following supporting information can be downloaded at https://www.mdpi.com/article/10.3390/nano15161252/s1. Figure S1. Graphical representation of the reduction rates with respect to the reaction temperature. Figure S2. Carbon contents of glycol-derived samples; Figure S3. Wt.% of the proportion of the prepared samples. Figure S4. Calculated specific capacity values from the CV and GCD results. Figure S5. The contribution of the diffusion-controlled capacity and diffusion-independent capacity at 10 mV/s: (a) EV (b) DV (c) TV and (d) TTV. Figure S6. Initial and final cycles of the prepared samples: (a) EV, (b) DV, (c) TV and (d) TTV. Figure S7. The cyclic stability of the DV//AC electrode for 2000 cycles. Figure S8. (a) XRD patterns of the DV electrode pre and post cycling. (b) Enlarged view. Figure S9. Coated DV electrode at (a–c) different magnifications. Figure S10. Cycled DV electrode at (a–c) different magnifications. Figure S11. Energy and power performance of the EV//AC, DV//AC, TV//AC and TTV//AC asymmetric devices compared with the reported studies.
