# Supplementary material for: Liquid-Phase Synthesis of Monodispersed V5+ Faradic Electrode Toward High-Performance Supercapacitor Application
Source: Nanomaterials (Basel). 2025 Aug 14;15(16):1252. doi: 10.3390/nano15161252 (PMC12388273; doi:10.3390/nano15161252)
Supplement: Supplementary file 1 [file nanomaterials-15-01252-s001.zip › nanomaterials-3788454-supplementary.pdf]

# Liquid phase synthesis of monodispersed $V^{5+}$ faradic electrode towards high performance supercapacitor application

**Sutharthani Kannan,[1,†] Chia-Hung Hong,[2,†] Pradeepa Stephen Sengolammal,[1] Suba Devi Rengapillai,[1,*] Sivakumar Marimuthu[1,*] and Wei-Ren Liu,[3,*]**

[1] #120, Energy Materials Lab, Department of Physics, Science Block, Alagappa University, Karaikudi-630003, Tamil Nadu, India.;susiva73@yahoo.co.in (SM), susimsk@yahoo.co.in (SDR), pradeepastephen8497@gmail.com (PSS) & sutharthanikannan26@gmail.com (SK)

[2] Department of Electrical Engineering, National University of Tainan, No. 33, Sec. 2, Shulin St., West Central District, Tainan City 700, Taiwan; chiahung@mail.mirdc.org.tw (CHH)

[3] Metal Industries Research and Development Centre, Kaohsiung 81160, Taiwan; chiahung@mail.mirdc.org.tw (CHH)

[4] Department of Chemical Engineering, R&D Center for Membrane Technology, Chung Yuan Christian University, 200 Chung Pei Road, Taoyuan 32023, Taiwan, ROC; wrliu@cycu.edu.tw (WRL)

* Authors to whom correspondence should be addressed.

† These authors contributed equally to this work.

**Copyright:** © 2025 by the authors. Submitted for possible open access publication under the terms and conditions of the Creative Commons Attribution (CC BY) license (https://creativecommons.org/licenses/by/4.0/).

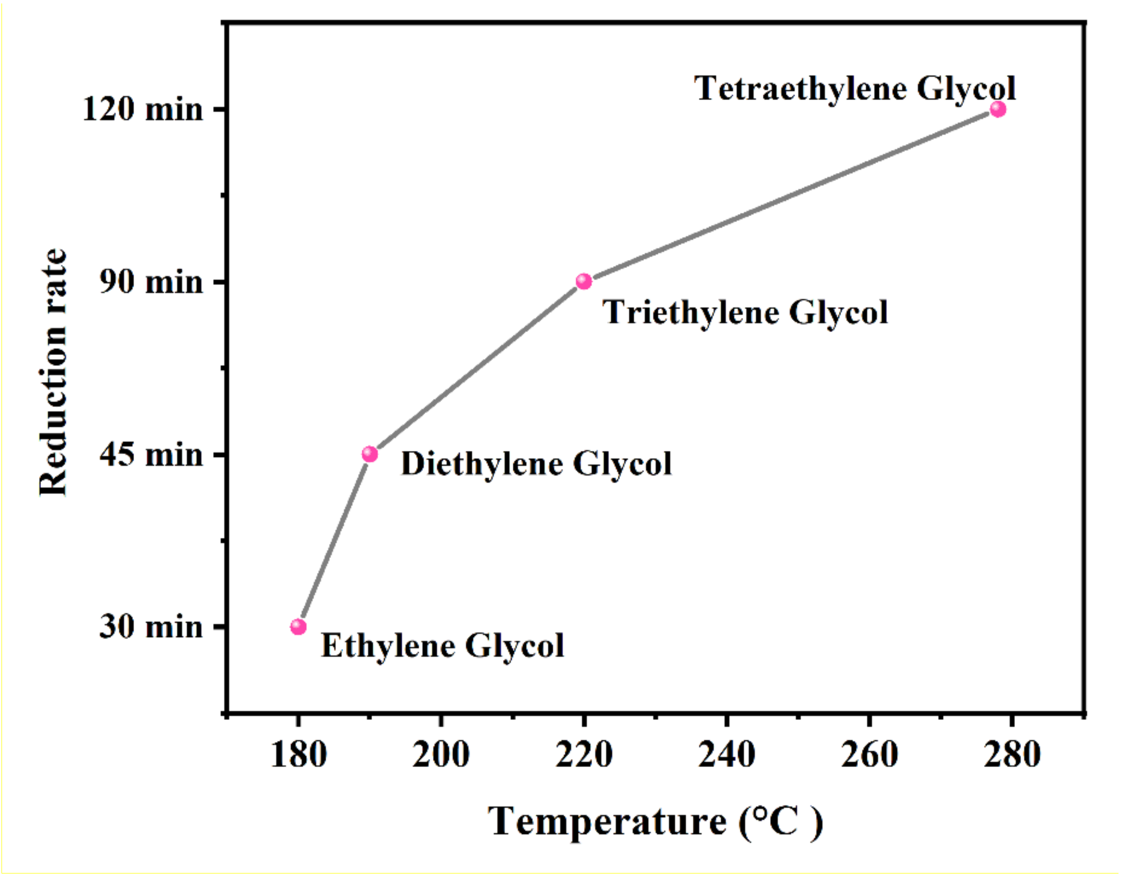


**Figure. S1**. Graphical representation of the reduction rates with respect to reaction temperature.

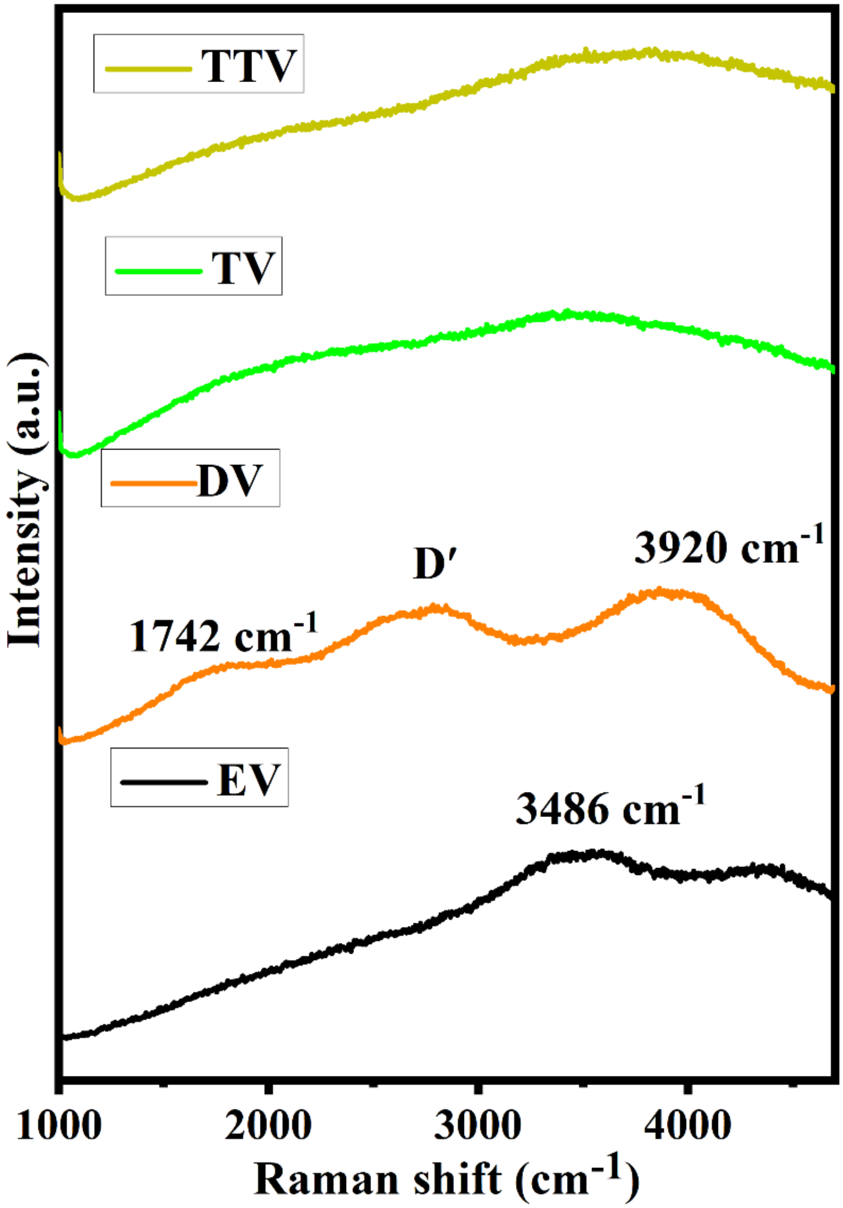


**Figure. S2.** Carbon contents in glycol derived samples.

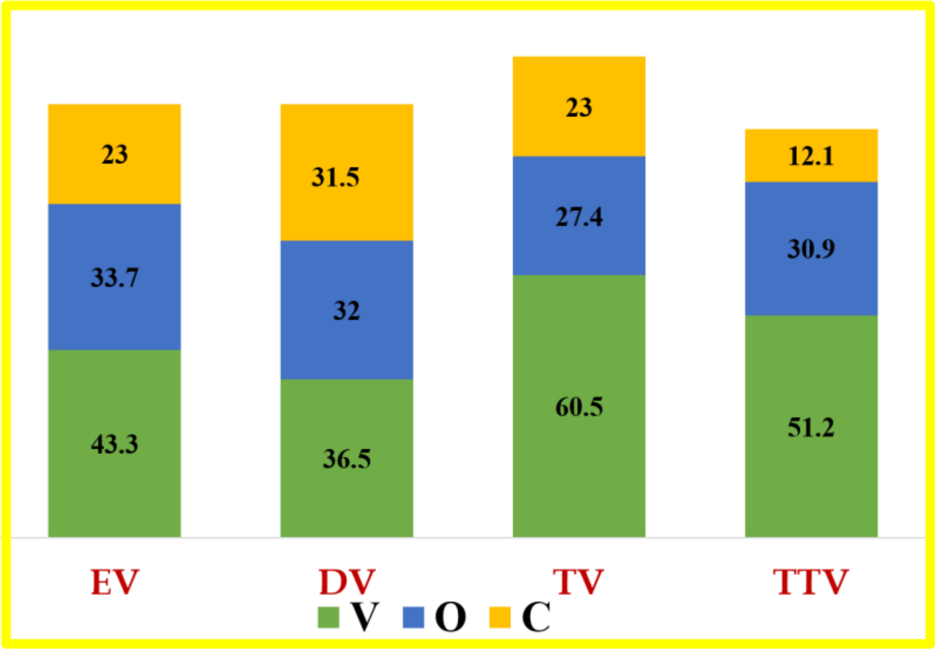


**Figure.S 3.** Weight % of the proportion of prepared samples.

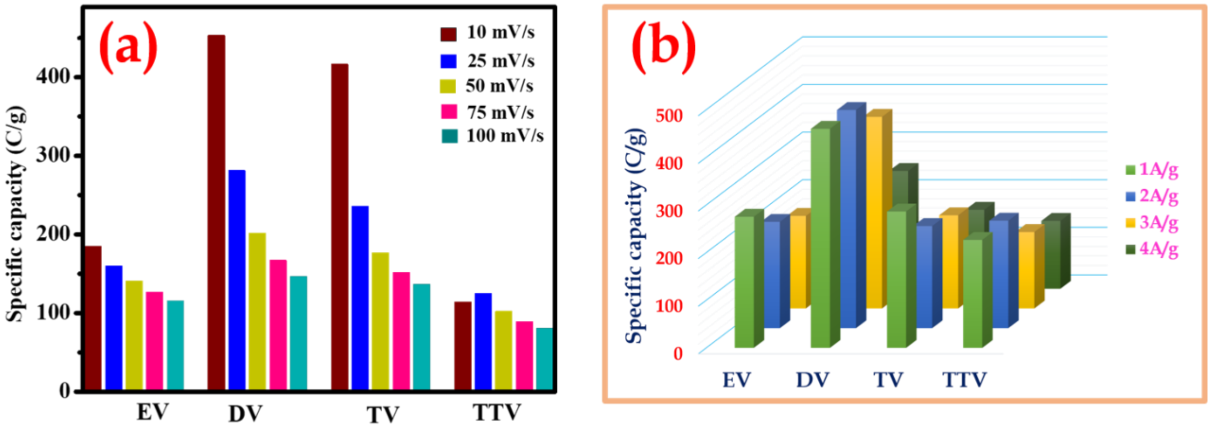


**Figure. S4.** Calculated specific capacity values from CV and GCD results.

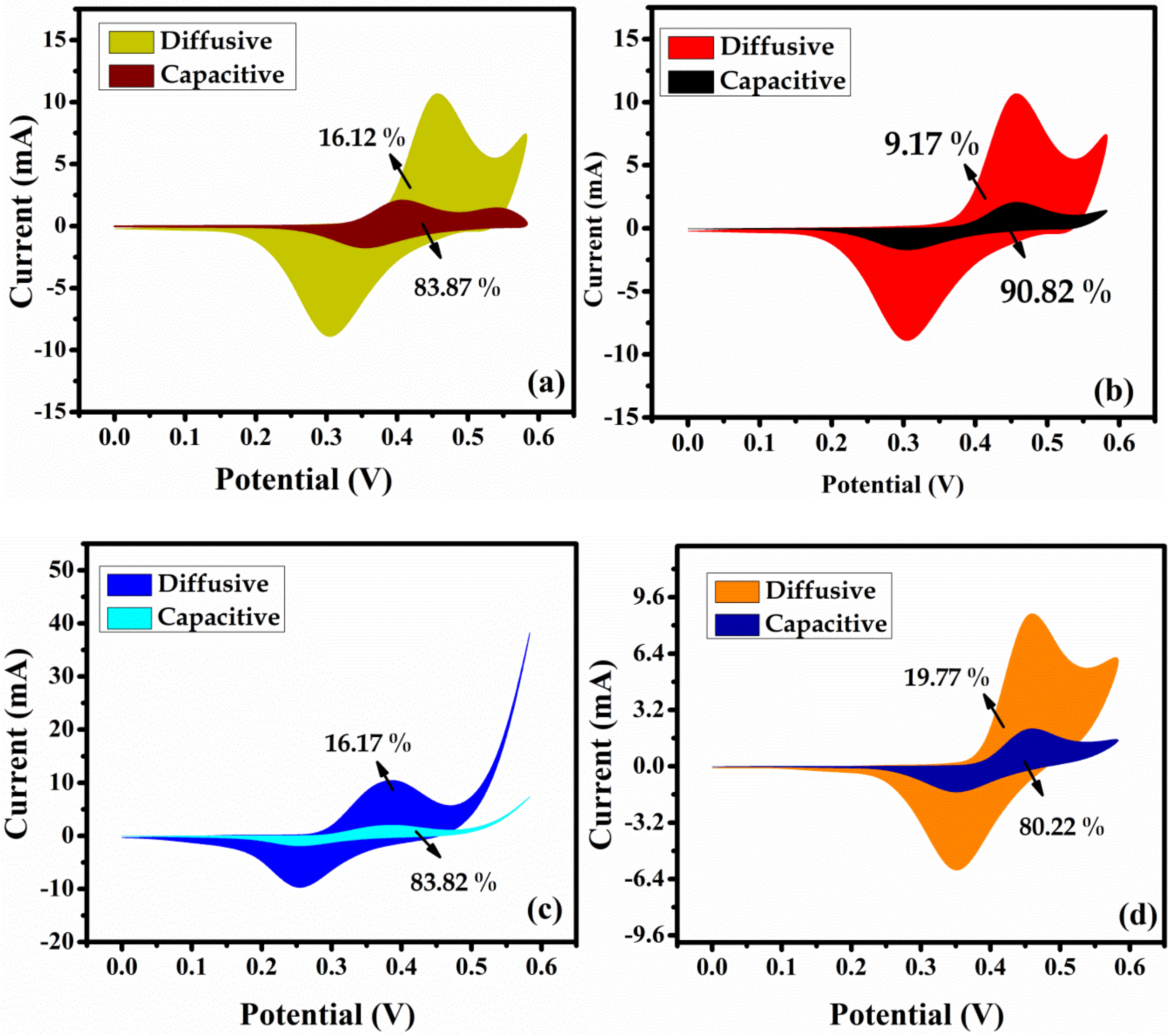


**Figure. S5.** The contribution of diffusion-controlled capacity and diffusion independent capacity at 10mV/s (a) EV (b) DV (c) TV and (d) TTV.

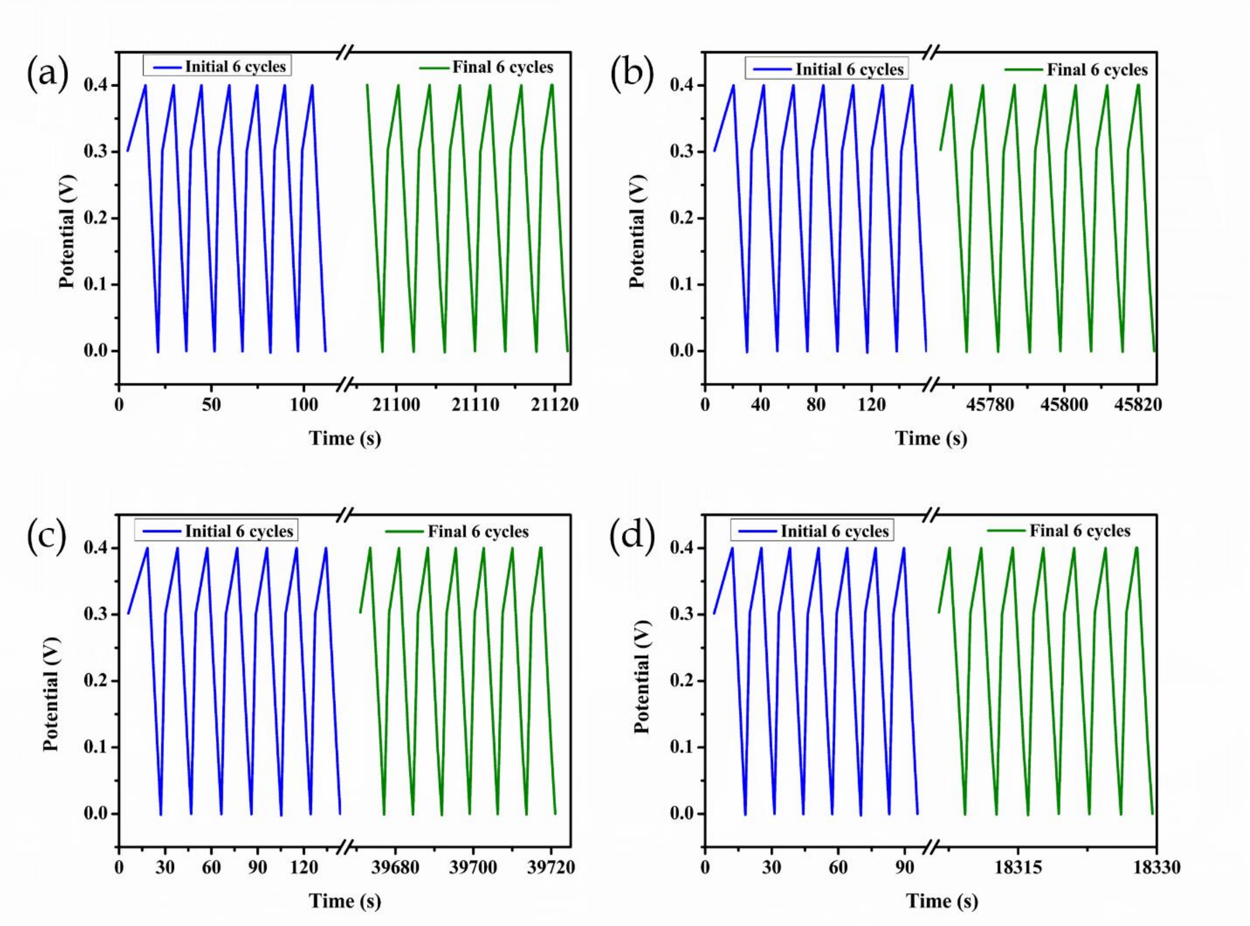


**Figure S6**. Initial and final cycles of prepared samples (a) EV, (b) DV, (c) TV and (d) TTV.

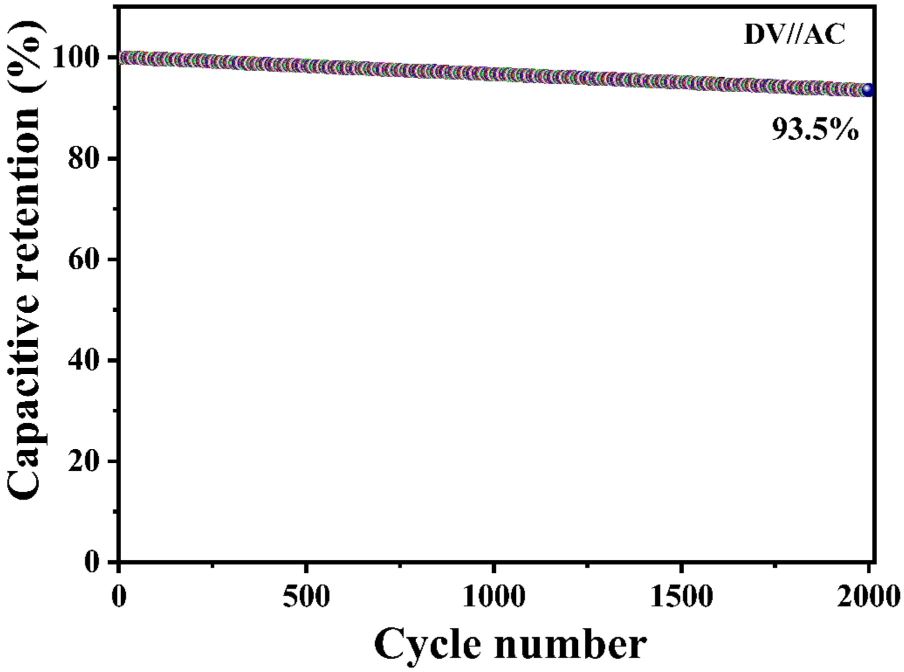


**Figure S7**. The cyclic stability of DV//AC electrode for 2000 cycles.

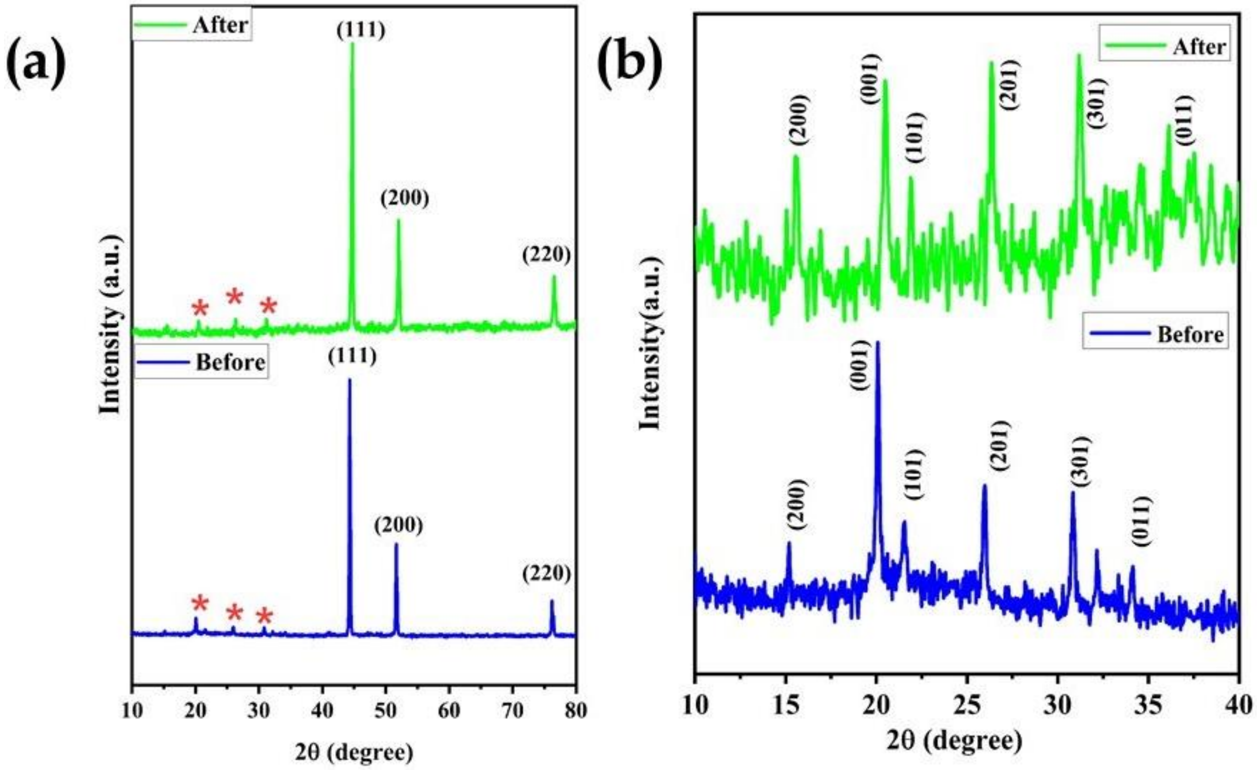


**Figure S8.** (a) XRD patterns of DV electrode pre and post cycling (b) Enlarged view.

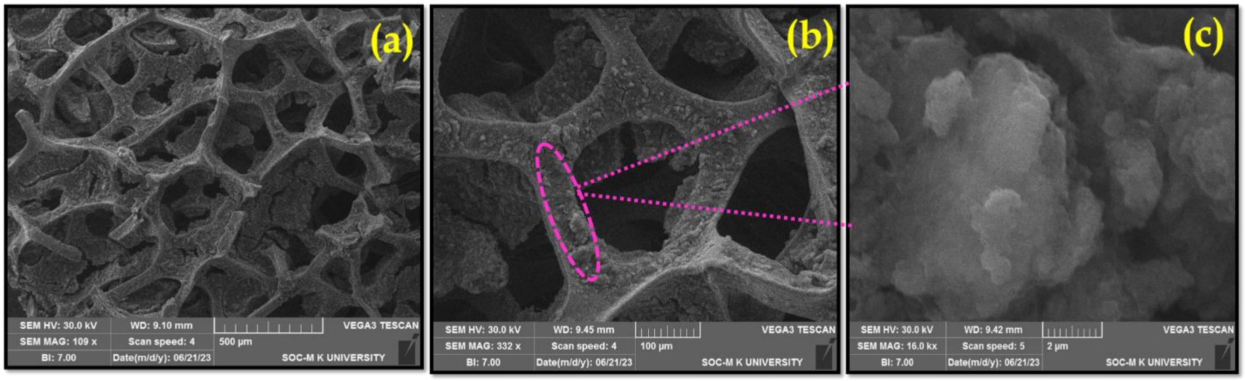


**Figure S9**. Coated DV electrode at (a-c) different magnifications.

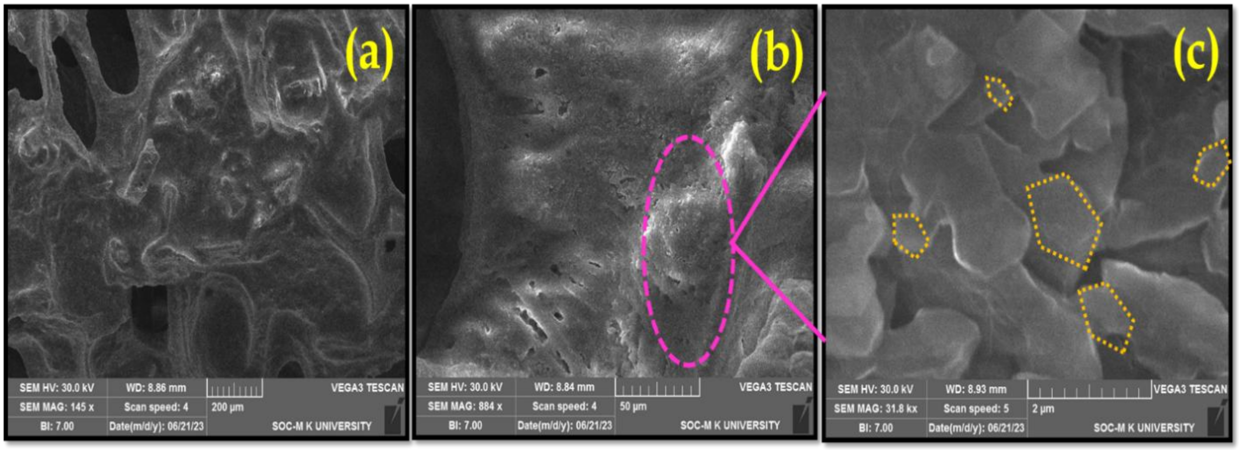


**Figure S10**. Cycled DV electrode at (a-c) different magnifications.

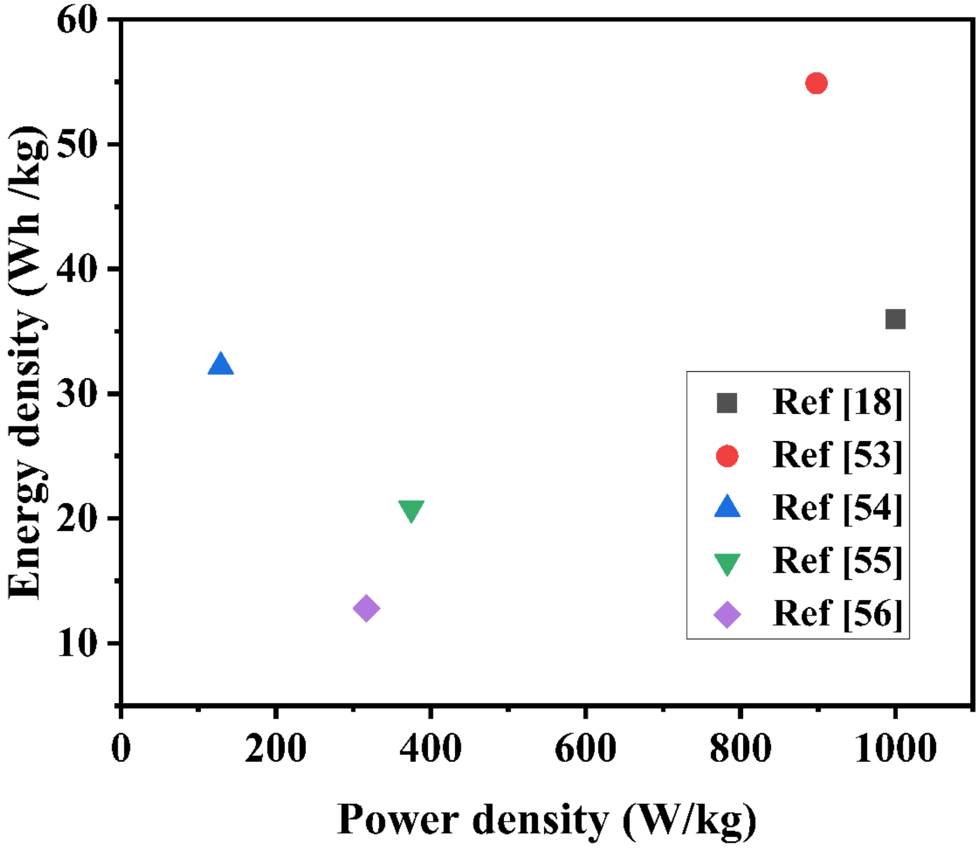


**Figure. S11** Energy and power performance of EV//AC, DV//AC, TV//AC and TTV//AC asymmetric devices compared with the reported studies.
